# Dynamic and heterogeneous impacts of granting and revoking elective c-section rights in São Paulo

**DOI:** 10.1093/heapol/czag021

**Published:** 2026-02-17

**Authors:** Gustavo Cordeiro, Judite Gonçalves, Mylene Lagarde

**Affiliations:** São Paulo School of Business Administration, Getulio Vargas Foundation (FGV), Rua Itapeva, 474, São Paulo 01332-000, SP, Brazil; Research Department, Institute for Health Policy Studies (IEPS), Rua Itapeva, 286, São Paulo 01332-000, SP, Brazil; Department of Primary Care and Public Health, Public Health Policy Evaluation Unit, School of Public Health, Imperial College London, 90 Wood Lane, London W12 0BZ, United Kingdom; Department of Health Policy, The London School of Economics and Political Science, Houghton Street, London WC2A 2AE, United Kingdom

**Keywords:** caesarean section, maternal health policy, health system equity, healthcare costs, natural experiment, synthetic control

## Abstract

Elective caesarean sections (c-sections) present a significant public health challenge due to associated health risks and increased costs. This study examines the causal impacts of a unique natural experiment in São Paulo, Brazil: Law 17,137/2019, which temporarily allowed pregnant women to opt for c-sections in public healthcare facilities. Using a difference-in-differences estimator, we analyse the Law's effects on c-section rates across various hospital types, municipal characteristics, and demographics. The Law led to a significant and immediate 3.03% point increase in c-section rates in public hospitals. Notably, this effect was limited to the public sector, with no consistent changes observed in private or mixed facilities. The impact was also temporary; following the Law's revocation less than a year later, c-section rates promptly reverted to pre-enactment levels, indicating no lasting effects. We find no evidence that the Law shifted deliveries from paid private care to free public hospitals. Our analysis reveals heterogeneous impacts, with the largest increases in c-section rates occurring in municipalities that had lower baseline c-section rates, a greater reliance on public healthcare, and fewer healthcare resources. These findings suggest that the law disproportionately affected areas with greater public health system strain. Interestingly, the increase in c-sections primarily occurred among low-risk births and had no detectable effect on newborn health outcomes, such as birth weight or Apgar scores. The additional 4500 c-sections performed under the law created an added fiscal burden of approximately R$459 000 for the public health system, based on the cost difference between vaginal and c-section deliveries. This study underscores that while granting elective choice may seem empowering, it can lead to a surge in unnecessary, costly, and riskier procedures, highlighting the crucial need to consider both equity and resource implications when designing healthcare policies.

KEY MESSAGES

- Granting pregnant women the option to choose an elective c-section in São Paulo, Brazil, significantly increased c-section rates. This policy, Law 17,137/2019, resulted in a notable 3.03% point increase in c-section rates in public hospitals immediately after implementation.
- The increase in c-sections was exclusively driven by public hospitals, with no consistent changes observed in private or mixed-facility c-section rates. The Law's effects were temporary, as rates returned to baseline levels after the Law was revoked in less than a year.
- The impacts were heterogeneous, being most pronounced in municipalities with lower baseline c-section rates, a greater reliance on public healthcare, and fewer public healthcare resources, such as hospital beds. This suggests that the Law had a greater effect in areas where the public system was already under more pressure and where women may have had fewer options.
- The increase in c-sections primarily occurred among low-risk births and had no detectable effect on newborn health outcomes, such as birth weight, gestational weeks, or Apgar scores. While the Law did not improve health metrics, the additional 4500 c-sections performed during its effect created an added fiscal burden of approximately R$459 000 for the public health system, based on the reimbursement difference between vaginal and c-section deliveries.

## Introduction

Caesarean section (c-section) delivery is unnecessary when a safe vaginal birth is possible. Despite its higher costs and associated health risks for both mothers and babies, especially in low-resource settings, the global c-section rate has nearly doubled since 2000, exceeding 20% of live births in 2015 (Rogers et al. 2017, Boerma et al. 2018, Fobelets et al. 2018, Sandall et al. 2018, Negrini et al. 2021). Evidence suggests that c-section rates well above 10%–19% are not associated with further reductions in maternal or neonatal mortality, highlighting substantial overuse in many settings (Betran et al. 2015, Molina et al. 2015). In Brazil, a country with one of the highest c-section rates in the world, the overall c-section rate exceeds 55%—especially high in private hospitals, when there is no obstetric indication for c-section, and among more educated mothers (Boerma et al. 2018).

Unnecessary c-sections are performed for several reasons associated with childbearing women’s preferences and beliefs (e.g. fear and anxiety, prior negative delivery experiences), health professionals’ preferences (e.g. convenience, financial reward), and health systems and organizations’ characteristics (e.g. reimbursement schemes, organizational culture) (Betrán et al. 2018). From a health economics perspective, the choice of delivery mode reflects the interaction between demand-side factors (women’s preferences, beliefs, and information), supply-side incentives (physicians’ time costs and convenience), and institutional constraints (clinical protocols, legal rules, and system capacity). A large literature shows that physician behaviour and organizational context can substantially influence intervention rates even in the absence of clear clinical indications (Gruber and Owings 1996, Currie and MacLeod 2008, Currie et al. 2024).

Various policies and interventions across countries and settings—including in Brazil—have aimed to reduce c-section rates, with some focusing on demand-side incentives and enhancing women’s autonomy (Massavon et al. 2017, Betrán et al. 2018, Yu et al. 2019, de Loenzien et al. 2021, Melo and Menezes-Filho 2023), and others targeting providers through regulation or financial incentives (e.g. Pilvar and Yousefi 2021). A review of non-clinical interventions to reduce unnecessary c-sections found that those targeting healthcare professionals are most effective, compared to those targeting women or healthcare facilities alone (Chen et al. 2018). Recent causal evidence from Brazil suggests that the way women’s autonomy is framed—either as informed choice or as an unconditional right—can lead to markedly different behavioural responses (de Oliveira et al. 2022, Melo and Menezes-Filho 2023).

Conceptually, demand-side autonomy-enhancing policies may operate through at least two distinct mechanisms. First, autonomy framed as informed choice—through counselling, disclosure of risks and benefits, and consent requirements—may reduce unnecessary c-sections by correcting misperceptions and empowering women to opt for vaginal delivery when clinically appropriate (Melo and Menezes-Filho 2023). Second, autonomy framed as an entitlement to elective c-section may instead increase utilization by lowering institutional and psychological barriers to requesting surgery, particularly in zero-price public systems where prior gatekeeping was binding (de Oliveira et al. 2022). These mechanisms generate opposite predictions for c-section rates and help explain heterogeneous effects of previous autonomy-enhancing policies.

In Brazil, these conceptual distinctions are particularly salient given longstanding differences between the public and private sectors in childbirth-related decision-making. Recent evidence shows that both women and physicians have substantially less influence over delivery-related medical decisions in the public sector than in the private sector, where greater discretion and stronger financial and organizational incentives prevail (Spinola and Rocha 2025). This institutional asymmetry has contributed to stark differences in delivery practices across sectors (Context section) and provides a rationale for policies designed to equalize women’s autonomy between public and private care, including the one evaluated here.

In this study, we analyse a legal change in Brazil that framed elective c-section as an entitlement. On 23 August 2019, the legislative assembly of São Paulo, a state with over 44 million inhabitants, passed Law 17,137, allowing pregnant women to opt for c-section delivery even if there was no medical necessity. The Law reserved the option to women in at least their 39th week of gestation and mandated that they were informed of the benefits of vaginal delivery and the risks of c-section delivery. The Law also reserved the possibility of referral to another professional when the attending doctor disagreed with the pregnant woman’s choice. Lastly, additional expenses were to be accommodated by the hospital’s budget. While the Law applied to both public and private providers, it primarily altered the institutional constraints faced by women giving birth in the public sector, as elective c-sections were already widely available in private hospitals prior to the Law.

Less than 1 year later, in July 2020, Law 17,137/2019 was deemed unconstitutional and revoked, since the Federal Constitution of Brazil states that women can have a c-section delivery only when medically justified. This unique shock—a law allowing women to choose c-section and then removing that option—created a natural experiment for examining how policy changes affecting women’s autonomy influence their choice of mode of delivery. Importantly, the Law’s introduction and subsequent withdrawal allow us to distinguish between short-run behavioural responses to expanded choice and potential persistence through habit formation or path dependence. Prior work shows that increases in primary c-sections are particularly consequential, as they raise the likelihood of repeat c-sections in subsequent births (MacDorman et al. 2008, Betrán et al. 2018) making even temporary shocks potentially relevant for long-term system dynamics.

In this study, we examine the causal effects of introducing and subsequently revoking an entitlement to elective c-section in São Paulo, Brazil. Using individual-level birth records from the Brazilian Registry of Live Births linked to hospital and municipal characteristics, we implement a difference-in-differences design using the doubly robust estimator proposed by Callaway and Sant’Anna (2021). Our analysis proceeds in several steps. First, we estimate the dynamic effects of Law 17,137/2019 on c-section rates in São Paulo relative to other Brazilian states, distinguishing between births occurring in public, private, and mixed hospitals. Second, we assess whether the expansion of elective choice affected the sector in which women gave birth, by analysing shifts between public and non-public facilities. Third, we examine whether changes in delivery mode translated into short-run health consequences, considering standard neonatal outcomes recorded at birth as well as maternal and infant mortality and infant hospitalizations within 30 days. Finally, we explore heterogeneity in policy impacts across clinical risk groups, baseline c-section rates, and municipal socioeconomic and health system characteristics, including reliance on the public sector and local healthcare capacity.

Our work contributes to the literature in several ways. First, we build on de Oliveira et al. (2022) —who studied the overall, short-run impact of Law 17,137/2019— by examining the impact of the Law on different types of hospitals (i.e. public, mixed, private) and by using the Law’s revocation to distinguish short-run behavioural responses from potential persistence beyond the policy period. Similar to Melo and Menezes-Filho (2023) and Melo (2021), we analyse the causal impact of a demand-side intervention affecting women’s autonomy in childbirth; however, the two interventions differ fundamentally in their framing and institutional content. Those studies evaluate a nationwide resolution issued by the Brazilian Federal Council of Medicine (Resolution 2144), which restricted elective c-sections before 39 weeks of gestation and was accompanied by a strong informational and consent-based component aimed at promoting informed choice, implemented in 2016. Our study differs in three key aspects: (i) Law 17,137 was limited to one state, hence creating a natural experiment, and most importantly, it was legally binding; (ii) whereas Resolution 2144 combined a gestational-age restriction with an informed-consent framework, Law 17,137 explicitly framed elective c-section as a legal entitlement in the public sector after 39 weeks, without altering clinical guidelines or provider incentives; (iii) Law 17,137 was revoked after less than a year, allowing us to investigate the effects of eliminating that option, namely if any persistent effects remained.

Second, we explicitly test whether expanding entitlement to elective c-section in the public system induced substitution from paid private care towards free public provision. While concerns about moral hazard and crowd-out are central to debates on expanding publicly financed healthcare entitlements (Gruber and Simon 2008, Einav and Finkelstein 2018), empirical evidence on sectoral substitution in maternity care remains limited.

Third, we add to a literature that has predominantly focused on policies aimed at reducing caesarean section rates by examining the health consequences of a policy that increased elective c-sections. While existing studies largely evaluate supply- or demand-side interventions designed to curb overuse (Chapman et al. 2019, Yu et al. 2019, Melo and Menezes-Filho 2023), much less is known about the health implications of policy-induced increases in c-section utilization.

Fourth, we add to the health economics literature on (public vs private) healthcare provision by examining how institutional and capacity-related characteristics at the municipal level (e.g. rates of hospital beds and healthcare staff) condition the effects of autonomy-enhancing policies.

### Context

**Figure 1:**
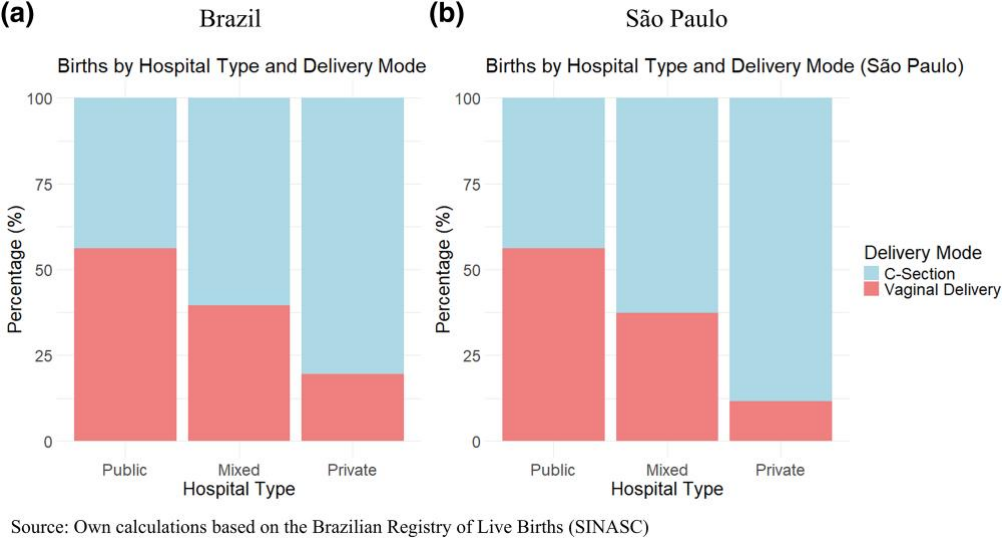
Prevalence of delivery mode per hospital type.

In Brazil, almost all births take place in the hospital. Brazil’s Unified Health System (Sistema Único de Saúde, SUS) provides universal health coverage, including prenatal, delivery, postpartum, and neonatal care, free of charge. Additionally, the private sector caters to privately insured and out-of-pocket paying individuals, and there are also private providers that reserve some capacity for SUS users (‘mixed’ units). In 2018, SUS hospitals accounted for 45.5% of all births, private hospitals for 19%, and mixed hospitals for 35.5%. Figure 1 presents the prevalence of each delivery mode per hospital type for the entire country and only for the state of São Paulo.

The SUS reimburses public and mixed hospitals per procedure, according to a predefined schedule. In 2021, the reimbursement amounts for regular vaginal and c-section deliveries were R$443 and R$545, respectively (SIGTAP 2025). The difference is not substantial and has not been identified as a strong determinant of c-section rates (Melo and Menezes-Filho 2023). Most obstetricians in the SUS are salaried and have no financial incentives to perform a c-section over vaginal delivery. In contrast, in the private sector, hospitals may charge varying amounts directly to patients or insurers, and obstetricians may either be salaried or paid per procedure. Thus, financial incentives may play a role in opting for c-section delivery, in addition to the greater convenience (ability to plan and adapt to a convenient work schedule and less time required).

São Paulo’s Law 17,137/2019 followed from national-level Resolution 2144 passed by the Federal Council of Medicine in 2016. This Council regulates medical practice in Brazil. Resolution 2144/2016 stipulated that from the 39th week of gestation, a pregnant woman has the right to opt for c-section delivery as long as she is informed of the risks and benefits of alternative delivery modes and signs an informed consent. This Resolution was not legally binding but had a strong framing around information and awareness. Melo and Menezes-Filho (2023) estimated that the passing of Resolution 2144/2016 reduced c-section rates by 1.6% points, probably because some less educated women from lower socioeconomic backgrounds—who are attended by the SUS—gained awareness of the risks and benefits of vaginal and c-section delivery and opted for vaginal delivery, especially when those women had previously delivered by c-section.

Private hospitals did not contribute to the estimated reduction in c-section rates in Melo and Menezes-Filho (2023), possibly because pregnant women attended in the private sector may have stronger preferences for convenience and/or be more easily persuaded by their doctor to opt for c-section (these women are usually assisted by the obstetrician who followed them during the pregnancy). This inequality, whereby women who can afford private care may choose their mode of delivery while poorer women attended by public hospitals may not, prompted São Paulo’s Law 17,137/2019. This Law, despite not being restricted to any specific type of hospital, forced public hospitals in São Paulo to oblige to each pregnant woman’s choice. It was framed around entitlement.

Law 17,137/2019 not only made c-section more accessible in unnecessary cases in the state of São Paulo, making it easier to accommodate women’s and health professionals’ preferences for this mode of delivery, but also validated those preferences and potentially reinforced wrong beliefs that c-section is safer. For example, a draft version of Law 17,137/2019 argued that in public hospitals, many women requesting c-sections are made to ‘suffer long hours to deliver vaginally’ and questioned the scientific evidence around mode of delivery and maternal and infant outcomes. De Oliveira et al. (2022) estimated a 3% increase in the overall c-section rate when Law 17,137/2019 was passed in São Paulo compared to other states. This is a meaningful increase given the already high c-section rates in Brazil—and in São Paulo specifically—and given that the study pools together all hospitals and not just public ones, where c-section rates are much lower. Additionally, Law 17,137/2019 may have shifted some c-sections from the private to the public sector to avoid out-of-pocket costs. This effect is likely small because pregnant women from higher socioeconomic status will tend to prefer to be attended by the doctor who followed them during pregnancy. Yet, this potential impact is also worthy of investigation.

## Methods

### Data

Our dataset integrates information from multiple sources covering the period from 1 January 2017 to 31 December 2021. We gathered birth information from the Brazilian Registry of Live Births (SINASC), hospital and health workforce characteristics from the National Registry of Health Facilities (CNES), and municipal characteristics from the Brazilian Institute of Geography and Statistics (IBGE). In total, our sample includes 14,129,463 births from all 27 Brazilian states (Brazil has 26 states and one federal district, which for the sake of this project is considered a state as well).

Our primary outcome is an indicator for c-section delivery, defined as a binary variable equal to one if the birth occurred via c-section and zero otherwise. To examine whether the Law also affected where women gave birth, we additionally analyse the choice of delivery sector, defined as an indicator for delivery in a public hospital versus a non-public hospital (private or mixed). To assess whether changes in delivery mode translated into short-run health effects, we also analyse standard neonatal outcomes recorded at birth, including gestational length (in weeks), birthweight (in grams), and Apgar scores at one and 5 minutes after birth. Finally, to capture potential downstream health system consequences, we examine maternal mortality, 30-day infant mortality, and infant hospitalizations within 30 days of birth, aggregated at the municipality level. (Data on maternal and infant mortality came from the Mortality Information System and data on infant hospitalizations came from the Hospitalizations Information System. As there is no key allowing direct linkage with the Living Birth Registries dataset, we chose to work with these indicators aggregated at the municipal level.) These measures are routinely used in the obstetric and health economics literatures to capture immediate neonatal health and early postnatal outcomes associated with birth timing and obstetric intervention (Dahlen et al. 2014, Ye et al. 2016, Declercq et al. 2025, Kennedy-Moulton et al. 2022).

To account for clinical heterogeneity in obstetric risk, we use the Robson classification, which groups births into 10 mutually exclusive categories based on routinely collected obstetric characteristics (parity, previous c-section, onset of labour, gestational age, foetal presentation, and plurality). Although the Robson classification is not a clinical risk score, it provides a standardized framework for comparing c-section rates across populations and settings. Following the literature, we classify Robson groups 6–9 as high-risk and use this classification both as a stratification variable and as a proxy for medical indication in our heterogeneity analyses (Zeitlin et al. 2021, Ramos et al. 2022).

We further incorporate individual, facility, and municipal characteristics to examine whether the policy’s effects varied across socioeconomic and institutional contexts. Facility type either public, private, or mixed, reflecting differences in financing, management, and provider incentives. Individual-level covariates include maternal education, race, and marital status. At the municipal level, we include baseline c-section rates, economic development (GDP per capita), indicators of health system organization and capacity—including Family Health Strategy coverage, private health insurance coverage, public hospital beds per capita, and health workforce density. Together, these variables allow us to assess whether responses to the policy were shaped by pre-existing practice patterns, reliance on the public sector, and constraints in local healthcare capacity.

Supplementary Table A1 in Online Appendix A provides a detailed description of all variables and their theoretical motivations, while Supplementary Table A2 presents the descriptive statistics.

### Analyses

#### Main analyses

We employ a difference-in-differences estimator to evaluate the impact of Law 17,137/2019. Our analysis proceeds in four steps: (i) a replication of the aggregate effects documented by de Oliveira et al. (2022); (ii) an assessment of whether the Law affected the choice of delivery sector (public versus non-public); (iii) an examination of c-section rates by hospital type (public, mixed, and private); (iv) an evaluation of the Law’s impacts on neonatal outcomes; and (v) an exploration of effect heterogeneity across clinical, and institutional, municipal contexts.

The estimator is the doubly-robust estimator proposed by Callaway and Sant’Anna (2021). Although our treatment is not staggered, this framework is preferable to standard Two-Way Fixed Effects for identifying causal treatment effects, as it accounts for potential misspecification in either the outcome regression or the propensity score models. Throughout, we report dynamic aggregation results in the form of event-study-type plots, which trace the average treatment effect of the Law in each bimester relative to its enactment.

Identification relies on the conditional parallel trends assumption, implying that in the absence of the Law, outcomes for births in São Paulo would have followed the same trajectory as those in the control group (states other than São Paulo). Pretreatment coefficients provide a direct, partial assessment of this assumption.

Our primary specification is a parsimonious model without covariates. We choose this as the baseline to maximize statistical power and avoid the multicollinearity introduced when annual municipal indicators are included alongside municipality fixed effects. All models include time and municipality fixed effects, and standard errors are clustered at the municipality level to account for local correlation in birth outcomes.

Formally, we estimate the Group-Time Average Treatment Effect (ATT(g,t)) for each bimester *t*, where *g* denotes the treated group (state of São Paulo). The doubly robust ATT is defined as:


$$ATT_{\left(g,t\right)}=E\;[(\omega_{DR}^{TR}-\;\omega_{DR}^{CO})\Delta Y_{t}]$$

Where:

-
$\Delta Y_{t}$ is the change in the outcome of interest (e.g. whether newborn *i* in municipality *m* in bimester *t* was delivered by c-section) between time t and the reference period;
-
$\omega_{DR}^{TR}-\;\omega_{DR}^{CO}\;$ are doubly robust weights combining propensity score reweighting and outcome regression adjustments based on maternal and municipal characteristics .

#### Robustness checks

As a robustness check, we estimate models controlling for maternal and/or municipal covariates. We also conduct a placebo check by artificially shifting the treatment date to 1 year prior to the actual implementation of Law 17,137.

A potential threat to our identification strategy is the COVID-19 pandemic, which partially overlaps with the period during which Law 17,137 was in force. Our identification relies on the assumption that, absent the Law, outcomes in São Paulo would have followed parallel trends to those in the comparison states, and—at the individual level—that treated and control observations are comparable over time conditional on observed characteristics. We argue that COVID-19 poses a limited threat to these assumptions for four reasons. First, the main effects of the Law emerge in late 2019, prior to the onset of the pandemic: three of the five bimesters in which the Law was active precede the first confirmed COVID-19 cases in Brazil. Second, the doubly robust estimator of Callaway and Sant’Anna (2021) adjusts for differences in pre-treatment maternal and municipal characteristics, ensuring that treated and control units are comparable in terms of baseline socioeconomic conditions and health-system capacity that may shape vulnerability to the pandemic. Third, all specifications include municipal and time fixed effects, absorbing time-invariant local characteristics and common temporal shocks, including the nationwide progression of COVID-19. Finally, the revocation of the Law in July 2020 provides a falsification-type test. If differential pandemic exposure were driving the results, we would expect a persistent deviation in estimated effects after the pandemic onset. Instead, estimates return to baseline following the Law’s repeal—coinciding with the peak of the first pandemic wave—supporting the interpretation that the observed effects are attributable to the legislative change rather than to COVID-19-related shocks. Nevertheless, to formally address any remaining concerns regarding the pandemic's influence, we perform an additional robustness check where we include only observations prior to March 2020.

## Results

### Impact on overall c-section rates

**Figure 2:**
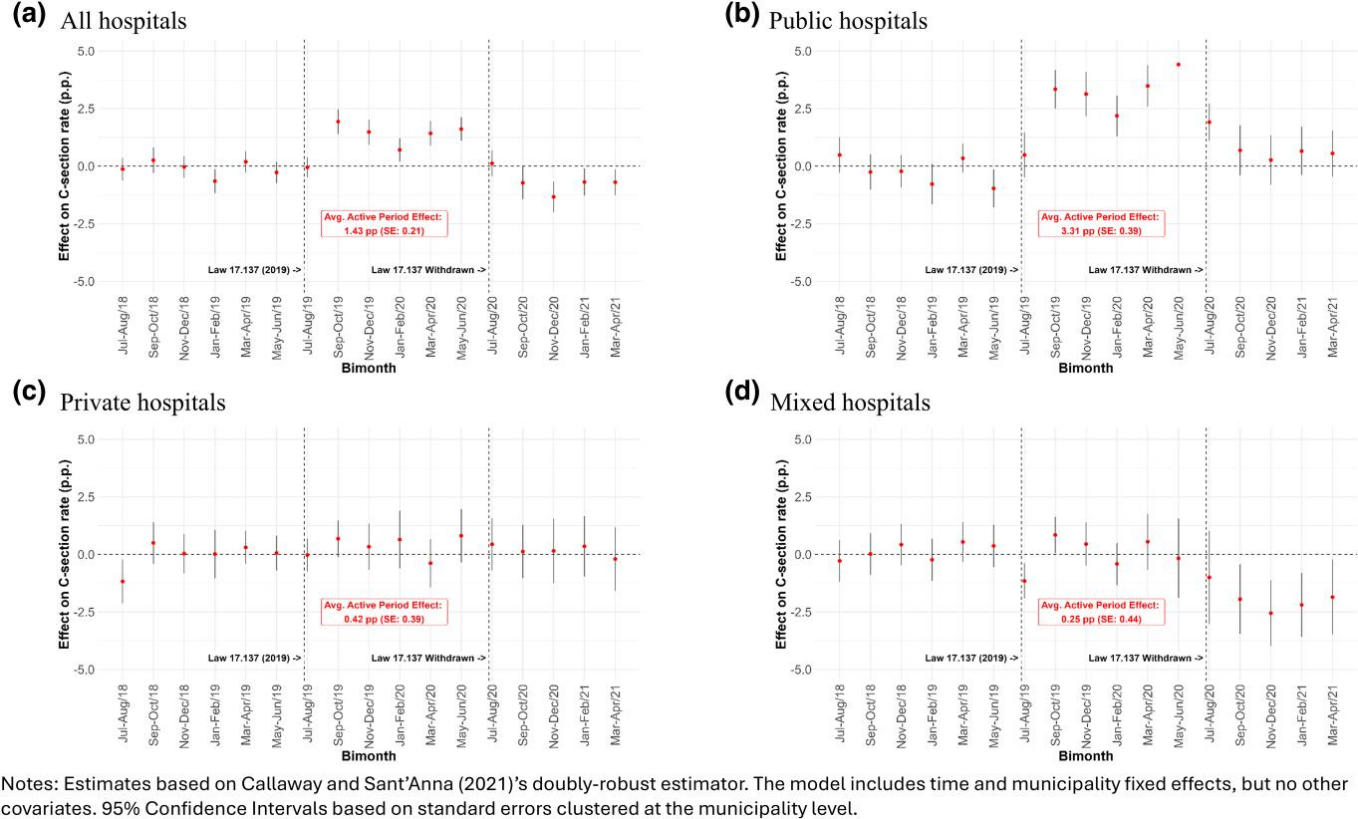
Effects of the passage and withdrawal of Law 17,137 on c-section rates.

The passage of Law 17,137 increased overall c-section rates by 1.43% points on average over the whole period the Law was in effect (SE = 0.21, *P* < 0.01), corresponding to a 2.4% increase relative to baseline rates (Fig. 2). Estimates for pre-Law periods are statistically indistinguishable from zero, supporting the parallel trends assumption. Following the Law’s revocation, c-section rates returned to baseline, indicating that the effect was temporary.

As a benchmark, de Oliveira et al. (2022) found 3% and 3.5% increases in c-section rates in the first and second bimesters following the passage of the Law (Sep-Oct and Nov-Dec 2019) —highly comparable to our estimates at 3.3% and 2.5% despite our different methodology and longer period of analysis.

As a robustness check, we re-estimate the model controlling for maternal and municipal characteristics (Supplementary Figure B1 - Appendix B). The estimated effects are very similar to the baseline specification. Adding maternal characteristics has virtually no impact on either the magnitude or precision of the estimates, consistent with the limited role of individual sociodemographic factors. By contrast, estimates become less precise when municipal covariates are included, with wider confidence intervals despite comparable point estimates. This likely reflects the limited within-municipality variation in these aggregate characteristics (measured annually) and their correlation with the time fixed effects and state-level policy timing.

Our identification strategy is further supported by two additional tests. First, a placebo test, conducted by artificially shifting the Law’s enactment date to 1 year prior (July-August 2018), shows no statistically significant effects (Supplementary Figure B2 - Appendix B). This reassures us that the observed results are not driven by pre-existing trends or anticipation of the Law. Second, to eliminate the confounding effects of the COVID-19 pandemic, we re-estimate the model excluding all observations post-March 2020. The results remain robust, with point estimates for the early active period of the Law identical to those obtained when using the full sample, suggesting that the pandemic was not a primary driver of the observed increase in c-section rates (Supplementary Figure B3 - Appendix B).

### Impact on choice to deliver in the public vs non-public sector

To assess whether expanded autonomy shifted delivery location, we estimate the probability that a birth occurred in a public hospital versus a non-public hospital (private or mixed) (Supplementary Appendix C). This directly tests whether women seeking elective c-sections substituted away from paid private care towards free public care following the Law’s enactment.

We find no statistically significant change in the likelihood of public versus non-public delivery over the period the Law was in force (average ATT = −0.37 pp, *P* > 0.1). Although a small, temporary shift—in the direction opposite to what could be expected—appears in the first post-Law bimester, it does not persist. Additional analyses decomposing non-public deliveries into private and mixed hospitals show no statistically significant shifts towards either type of facility. Overall, these results provide no evidence of systematic substitution across sectors.

### Impact on c-section rates by hospital type

Figure 2 also presents the impacts of the Law separately for births taking place in public, private, and mixed hospitals. The results reveal that the policy’s impact was heavily concentrated in public hospitals, where c-section rates increased by 3.31% points on average (SE = 0.37, *P* < 0.01), representing an over 8% increase from baseline c-section rates in these facilities. In contrast, private and mixed hospitals did not change their c-section rates after the enactment of the Law, with coefficients not statistically significant over the Law’s active period. Given this, all subsequent results pertain only to births in public hospitals.

Following the Law’s revocation, c-section rates in public hospitals returned to pre-treatment levels. In mixed hospitals, estimates show a modest post-revocation decline; however, there is no evidence of an increase during the Law’s active period, and this result should be interpreted with caution.

### Impacts on birth outcomes

The results for whether the policy-induced rise in c-section rates in public hospitals affected neonatal and maternal health are detailed in Supplementary Appendix D. Supplementary Figure D1 shows no detectable effects of the Law’s enactment on Apgar scores at 1 or 5 minutes in any period. Estimates are close to zero and precisely estimated, indicating no short-run neonatal health gains. Supplementary Figure D2 shows no consistent or sustained effects on birthweight or gestational length. While a small number of point estimates are weakly significant, the absence of a coherent pattern suggests no meaningful shift in delivery timing. Lastly, Supplementary Figure D3 shows no impacts on maternal mortality, 30-day infant mortality, or hospitalization within 30 days of birth (measured as municipality-level rates). Combined, these results suggest that the rise in elective c-section rates in public hospitals did not systematically compromise short-run neonatal outcomes.

### Heterogeneity analysis

We next examine whether the large effect observed for public hospitals varied across institutional and clinical contexts:

### Birth risk

**Figure 3:**
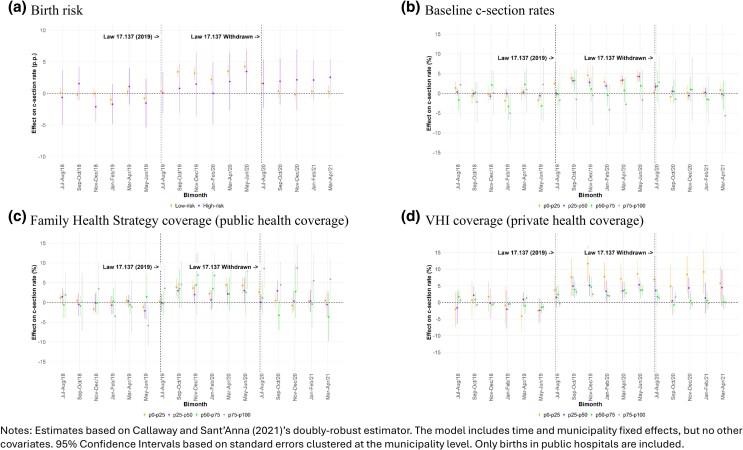
Heterogeneous effects of the passage and withdrawal of Law 17,137 on c-section rates.

Figure 3a shows the impact on c-section rates appears only among low-risk births. As expected, for high-risk births (Robson groups 6–9), the estimated impacts are not statistically different from zero; despite the wide confidence intervals, the point estimates are also considerably closer to zero compared to the estimates for the low-risk subsample. This is consistent with finding no impacts of the Law on neonatal outcomes.

### Baseline c-section rates

As shown in Fig. 3b, the impact on c-section rates was greater in municipalities with lower baseline c-section rates. Municipalities with baseline c-section rates in the first quartile (p0–p25) experienced increases in c-section rates of up to five percentage points while the Law was in place. Municipalities in the top two quartiles (p50–p75 and p75–p100) did not experience statistically significant impacts of the Law. (The quartiles are constructed at the municipality-level. As a result, at the individual level, the subsamples are not equally sized, and the sample sizes by quartile depend on the variable. Therefore, e.g. for baseline c-section rates, and given that we are focusing here on births in public hospitals, there are many more observations in lower quartiles, because in municipalities in the top quartiles, more births take place in the private sector. The different sample sizes are clear from the width of the confidence intervals in the figures.) Not only was there more room for c-section rates to increase where they were initially low, but also women in these municipalities may have faced more constraints on elective c-section before the Law, so expanding autonomy had a larger marginal impact (and the opposite for municipalities with already high c-section rates, which may have been already permissive).

### Health coverage

Finally, Fig. 3c and d illustrate that municipalities with higher Family Health Strategy coverage and lower voluntary health insurance coverage experienced greater impacts of the Law, albeit imprecisely estimated. Although here we are restricting the sample to births in public hospitals, Family Health Strategy and voluntary health insurance coverage are likely capturing the local health system context and how it mediates the impacts of the Law. For example, higher Family Health Strategy coverage could mean stronger ties between women and the public health system, increasing awareness of newly gained autonomy under Law 17,137. Municipalities with lower voluntary health insurance coverage likely have greater reliance on the public system, potentially amplifying the effect of a Law expanding women’s options within that system.

### Public healthcare resources

**Figure 4:**
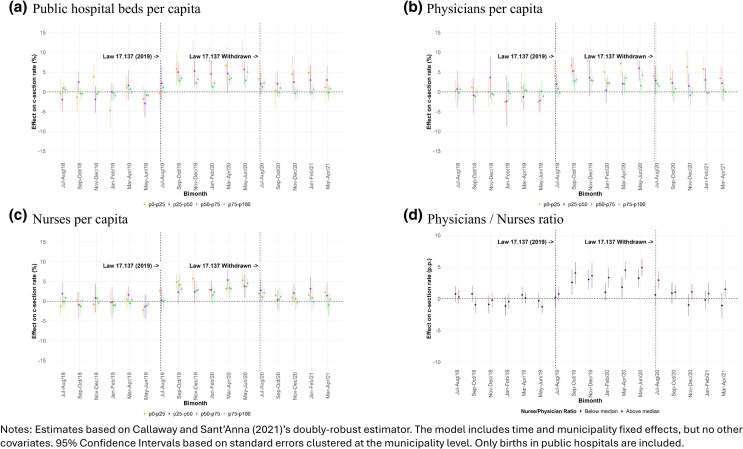
Heterogeneous effects of the passage and withdrawal of Law 17,137 on c-section rates by baseline healthcare resources.

Figure 4 shows that the Law led to greater increases in public c-section rates in municipalities with fewer hospital beds—less clear gradient by physicians and nurses per capita. Similar patterns were found when examining the ratio of obstetric nurses to gynaecologists/obstetricians. This could reflect supply-side bottlenecks: when women were given the option, demand for c-sections rose, especially where providers may not have previously offered elective c-sections due to resource constraints. The weaker gradient for physicians and nurses per capita suggests that facility capacity is more binding a constraint than availability of professionals.

### GDP per capita

The effect of the Law was not markedly different across municipalities with different GDP levels, as illustrated in Supplementary Appendix E, Figure E1, although municipalities in the top quartile display lower point estimates throughout the period when the Law was in place.

### Maternal education, race, and marital status

Supplementary Appendix E, Figure E2, presents results by demographic groups, showing similar impacts of Law 17,137 for all mothers regardless of their education level, race, or marital status. Across these groups, the effects appear broadly consistent, with no clear evidence of heterogeneity.

## Discussion

This study evaluates the short- and medium-term effects of Law 17,137/2019, a temporary policy in São Paulo that expanded maternal autonomy to choose c-section delivery in public hospitals without medical indication. Using a difference-in-differences design, we find that the Law increased c-section rates in public hospitals by over three percentage points (8% in relative terms), with no significant changes observed in mixed or private hospitals. The absence of a response in the private sector is consistent with the fact that women receiving private maternity care already faced few formal or informal barriers to electing c-section delivery prior to the Law. These effects were reversed upon the Law’s revocation, indicating no persistent shifts in delivery behaviour.

Our findings build on and extend those of de Oliveira et al. (2022), who documented the short-run, aggregate effects of the Law. We show that the increase was driven entirely by public hospitals, and that the Law’s revocation restored pre-policy c-section rates. Our results also complement Melo and Menezes-Filho (2023), who evaluated the impact of a non-binding national awareness policy. Unlike that soft intervention, Law 17,137 introduced a legally enforceable expansion of choice—limited to one state—which had immediate effects on behaviour. Taken together, these studies suggest that demand-side policies affecting women’s autonomy can have markedly different effects depending on whether autonomy is framed as informed choice (the case in Melo and Menezes-Filho 2023) or as an unconditional entitlement to elective intervention [de Oliveira et al. (2022) and our study].

A natural concern when expanding entitlement to elective caesarean delivery in the public system is that women may substitute away from paid private care towards free public provision, particularly when the policy relaxes previously binding access constraints. This shift from paid private care to free public provision, commonly referred to as the ‘crowd-out’ effect in health economics, has been well documented (Gruber and Simon 2008, Chen et al. 2023, Semprini 2023). However, we find no evidence that Law 17,137/2019 altered the likelihood of delivering in public versus non-public hospitals. This absence of sectoral substitution suggests that delivery location is shaped less by short-run financial incentives and more by pre-existing relationships with providers, continuity of antenatal care, and preferences for specific physicians—factors that are particularly salient in maternity care. As a result, the policy primarily affected delivery practices within the public sector rather than reallocating births across sectors.

From a theoretical perspective, the absence of health gains alongside increased utilization suggests that the marginal c-sections induced by the Law were of limited clinical value, aligning with evidence that c-section rates beyond recommended thresholds do not improve maternal or neonatal outcomes (Betran et al. 2015, Betrán et al. 2018). Most existing causal evidence on childbirth policy evaluates interventions designed to reduce caesarean section use, often motivated by concerns about overuse and cost containment (Chen et al. 2018, Melo and Menezes-Filho 2023). By contrast, far less is known about the consequences of policies that increase access to elective caesarean delivery. Our findings help fill this gap by showing that a policy-induced rise in c-sections—concentrated among low-risk births—did not generate short-run health benefits, nor detectable harms. This suggests that, in high–baseline-rate settings, marginal increases in elective caesareans are unlikely to improve clinical outcomes, reinforcing the view that utilization effects alone should not be interpreted as welfare gains. While short-run health outcomes were unaffected, increases in primary c-sections may nevertheless have longer-term implications given their strong association with repeat c-sections in subsequent pregnancies (MacDorman et al. 2008). However, our study period is too short to assess whether the increase in primary c-sections translated into higher rates of repeat c-sections in subsequent pregnancies, which may only materialize over a longer time horizon.

We further show that the Law’s impact was largest in municipalities with greater reliance on the public sector and more limited healthcare resources. These dimensions capture distinct but complementary mechanisms: public-sector dependence determines the degree of exposure to the policy, while resource constraints shape the magnitude of the response once procedural restrictions are relaxed. In municipalities where most births already occurred in public facilities, the Law applied to a larger share of deliveries, while tighter capacity and staffing constraints made previously binding organizational rules more salient, amplifying the effect of a sudden expansion in women’s procedural autonomy. The increase in c-section rates was driven by birth risk groups less often associated with indication for c-section per the Robson classification. This pattern is consistent with the view that the Law primarily relaxed previously binding organizational and procedural constraints in the public sector, rather than altering underlying maternal preferences or clinical need. Crucially, the increase in c-section rates had no detectable impacts on perinatal outcomes, including gestational weeks, birthweight, and Apgar scores.

Finally, we estimate that approximately 4500 additional c-sections were performed in public hospitals from the moment the Law was passed until when it was revoked. This number is calculated by applying the estimated treatment effect to the number of births in São Paulo while the Law was active (there were 316 919 births in public hospitals in São Paulo during the five bimesters of the Law’s operation; applying the estimated 1.43 percentage point increase leads to 4532 additional procedures). These additional C-sections generated an added fiscal burden of around R$459,000, based on the Brazilian Unified Health System reimbursement differential between vaginal and c-section deliveries. This value refers only to the amount transferred by the federal government to local government based on production; therefore, it is underestimated. While modest in aggregate, this figure highlights that even temporary expansions in delivery mode choice can produce measurable budgetary impacts in large-scale public health systems.

While our findings provide robust evidence of the direct effects of Law 17,137/2019, broader implications remain unexplored. First, the administrative data do not allow us to distinguish between maternal preferences and provider incentives as two key underlying mechanisms. Second, we only crudely capture potential downstream effects on maternal and infant morbidity, in the short run. Third, we do not assess potential indirect hospital- or system-level effects, such as the diversion of limited healthcare resources. In under-resourced public hospitals, even small increases in scheduled surgical deliveries may strain physical infrastructure (e.g. operating rooms, recovery beds) and human resources (e.g. anaesthesiologists, obstetricians), potentially crowding out care for more urgent cases (see e.g. Alexander and Currie 2017, on the role of capacity constraints in hospital admissions). Future work should explore these indirect consequences, particularly in settings where expanding choice must be balanced against service delivery constraints.

Although our analysis focuses on São Paulo and the Brazilian Unified Health System (SUS), the institutional features underlying our findings are shared by many publicly financed health systems internationally. In particular, our results are most relevant for settings in which maternity care is predominantly publicly funded, free at the point of use, and delivered in hospitals operating under fixed physical and human capacity constraints. This includes national health services and social health insurance systems in high-income countries (e.g. the United Kingdom, Spain, Portugal, and Italy), as well as middle-income countries with universal health coverage arrangements and mixed public–private provision (e.g. Mexico, Colombia, and Thailand). In such contexts, policies that expand legal entitlement to elective procedures—without accompanying changes in clinical guidelines or provider incentives—may similarly increase utilization and place pressure on already constrained public hospital resources.

Taken together, our findings offer novel evidence that increasing maternal autonomy in the public sector can shift delivery patterns towards larger c-section rates. These are largely unnecessary, costlier, and riskier procedures, underscoring the importance of considering both equity and resource implications when implementing autonomy-enhancing policies. Policies aimed at promoting autonomy in childbirth should therefore be carefully designed to align choice with evidence-based guidance and health system capacity, rather than relying on expanded entitlement alone.

## Supplementary Material

czag021_Supplementary_Data

## Data Availability

The data underlying this article were accessed from DATASUS (Departamento de Informática do SUS), the Brazilian Ministry of Health’s open-access data platform (https://datasus.saude.gov.br/). These data are publicly available and can be retrieved through the TABNET system or the FTP server (ftp://ftp.datasus.gov.br/). The derived data generated in this research will be shared on reasonable request to the corresponding author.

## Reflexivity statement

The authors include one male and two females and span different levels of seniority. One author, based in Brazil, has a background in management and specializes in health policy and economics, while the other two are senior researchers in the United Kingdom with expertise in health policy evaluation and global health systems. All three have experience researching the Brazilian health system, with a focus on health financing, equity, and service delivery.
