# Supplementary material for: Dynamic and heterogeneous impacts of granting and revoking elective c-section rights in São Paulo
Source: Health Policy Plan. 2026 Feb 17;41(4):650–60. doi: 10.1093/heapol/czag021 (PMC13135077; doi:10.1093/heapol/czag021)
Supplement: czag021_Supplementary_Data [file czag021_supplementary_data.docx]

**Dynamic and heterogeneous impacts of granting and revoking elective c-section rights in São Paulo**

**SUPPLEMENTARY MATERIALS**

Table of Contents

[Appendix A – Variable construction and summary statistics 2](#_Toc218711807)

[Appendix B – Robustness checks: alternative covariate sets 7](#_Toc218711808)

[Appendix C – Choice of hospital type for delivery 10](#_Toc218711809)

[Appendix D – Impacts on birth outcomes 12](#_Toc218711810)

[Appendix E – Heterogeneity by municipal characteristics and demographic characteristics of mothers 15](#_Toc218711811)

# Appendix A – Variable construction and summary statistics

Table A1 - Description of all variables and their theoretical motivation

| **Variable** | **Description** | **Theoretical justification** |
| --- | --- | --- |
| **Dependent variables** | | |
| **C-section** | Binary indicator of c-section (1) or vaginal delivery (0) | Captures utilization of a costly, invasive medical procedure that is sensitive to institutional rules, provider behaviour, and patient choice, making it the main outcome for evaluating the policy’s effect |
| **Public vs non-public delivery** | Indicator equal to one if the birth occurred in a public hospital and zero if it occurred in a non-public hospital (private or mixed) | Allows assessment of whether expanded autonomy shifted healthcare demand across sectors, testing substitution between public and private provision in response to changes in entitlement |
| **Apgar 1 / Apgar 5** | The Apgar is a test administered to newborns shortly after birth (at 1 and 5 minutes) that assesses heart rate, muscle tone, reflexes, respiration, and overall appearance. The Apgar score is measured on a scale from 0 to 10 and captures the newborn's overall health—the higher the score, the better | Standard short-run indicator of neonatal health, used to assess whether changes in delivery mode translate into clinically meaningful benefits or harms |
| **Gestational weeks** | The number of gestational weeks at birth | Sensitive to birth anticipation and scheduling, which may increase with elective c-sections; allows detection of changes in timing of delivery |
| **Birthweight** | The newborn’s weight, in grams | Proxy for foetal development and neonatal health; commonly affected by gestational length and obstetric intervention |
| **Maternal mortality ratio** | Number of maternal deaths per 100,000 live births at the municipal level | Captures severe maternal health consequences potentially linked to increased surgical delivery and obstetric complications |
| **Infant mortality (≤30 days)** | Number of deaths of children under thirty days of age per 1,000 live births at the municipal level | Standard early-life mortality measure used to assess short-run population health impacts of changes in obstetric care |
| **Infant hospitalization (≤30 days)** | Number of hospital admissions for infants under thirty days of age per 1,000 live births at the municipal level | Captures early postnatal morbidity and health system utilisation potentially affected by delivery mode |
| **Covariates & stratification variables** | | |
| **Robson classification (birth risk)** | The Robson classification groups women into ten mutually exclusive categories based on 6 basic obstetric indicators that are routinely collected in all maternities (parity, number of foetuses, previous c-section, onset of labour, gestational age, and foetal presentation). Although not a risk classification for use in practice—but rather a retrospective tool to analyse and compare c-section rates across different hospitals, regions, or countries (World Health Organization, 2019), we use it here as a proxy of birth risk. We consider groups 6–9 to be high-risk, based on empirical studies which have documented c-section rates close to 100% in these groups, including in São Paulo-based studies (Ramos et al., 2022; Zeitlin et al., 2021) | Allows distinction between clinically indicated and potentially discretionary c-sections, and tests whether the policy primarily affected low-risk births where medical necessity is limited |
| **Maternal characteristics** | - Marital status (single, married, other)  - Race (white, black, mixed, other)  - Education (0–7 years, 8–11 years, 12 or more years) | Capture socioeconomic gradients in access, preferences, information, and interactions with providers; used to test whether policy effects are heterogeneous across social groups |
| **Hospital type** | We classify hospitals as public, private, or mixed. Mixed hospitals cater to both public (SUS) and private patients; they are managed by private not-for-profit firms which reserve some capacity for SUS patients. Public hospitals are managed by federal, state, or municipal governments, and private hospitals are managed by private for-profit firms | Central to identifying institutional mechanisms: financial incentives, provider constraints, and patient choice differ sharply across hospital types, shaping responsiveness to the policy |
| **Municipal characteristics** | | |
| **Baseline c-section rate** | C-section rate in the year prior to the Law’s implementation | Captures pre-existing capacity, practice style, and institutional norms, which may condition responsiveness to policy shocks |
| **GDP per capita** | Municipal GDP per capita | Proxy for local economic development, healthcare demand, and supply capacity, potentially moderating policy effects |
| **Family Health Strategy coverage** | Percentage of the population covered by the Family Health Strategy (a flagship public primary healthcare program) | Reflects strength of primary care and continuity of care, which may influence referral patterns, birth planning, and reliance on hospital services |
| **Private health insurance coverage** | Percentage of the population with voluntary health insurance | Measures reliance on the private sector and potential access to alternative delivery options outside the public system |
| **Public hospital beds** | Rates of public hospital beds per 100,000 inhabitants | Proxy for physical hospital capacity, relevant for assessing whether increased elective procedures may strain resources |
| **Health workforce density** | Physicians / nurses (full-time equivalent) per 1,000 inhabitants. | Captures human resource constraints that may affect hospitals’ ability to absorb increases in scheduled surgical deliveries |

Table A2 – Descriptive statistics

|  | **São Paulo - Before** | **Other states - Before** | **São Paulo - After** | **Other states - After** | **p-value (SP Before vs OS Before)** |
| --- | --- | --- | --- | --- | --- |
| **Outcomes** |  |  |  |  |  |
| C-section rate | 0.59 (0.49) | 0.55 (0.50) | 0.59 (0.49) | 0.57 (0.50) | p<0.01 |
| Apgar 1 | 8.51 (2.84) | 8.41 (2.78) | 8.49 (2.78) | 8.41 (2.77) | p<0.01 |
| Apgar 5 | 9.53 (2.53) | 9.37 (2.35) | 9.51 (2.55) | 9.34 (2.25) | p<0.01 |
| Number of gestational weeks | 38.36 (2.13) | 38.51 (2.25) | 38.33 (2.14) | 38.48 (2.22) | p<0.01 |
| Birthweight | 3161.51 (557.91) | 3195.76 (562.04) | 3161.95 (561.65) | 3192.25 (565.23) | p<0.01 |
| Maternal mortality | 174.93  (886.06) | 133.11  (801.20) | 198.81  (890.03) | 172.81  (923.68) | p<0.05 |
| Infant mortality | 14.55  (28.11) | 10.77  (23.93) | 12.73  (27.36) | 10.15  (23.03) | p<0.01 |
| Infant hospitalization | 320.82  (418.74) | 183.05  (341.09) | 365.16  (501.14) | 206.32  (373.21) | p<0.01 |
| **Birth and mother characteristics** |  |  |  |  |  |
| Birth risk (Robson classification) |  |  |  |  |  |
| Low-risk | 93.80% | 90.80% | 93.60% | 91.50% | p<0.01 |
| High-risk | 5.80% | 5.50% | 6.00% | 5.40% |  |
| Unknown | 0.40% | 3.70% | 0.40% | 3.10% |  |
| Marital status |  |  |  |  |  |
| Single | 39.10% | 44.70% | 40.60% | 48.90% | p<0.01 |
| Married | 41.90% | 30.80% | 40.20% | 29.30% |  |
| Other | 19.00% | 24.50% | 19.10% | 21.70% |  |
| Race |  |  |  |  |  |
| White | 55.70% | 29.00% | 53.30% | 28.00% | p<0.01 |
| Black | 6.20% | 5.60% | 7.20% | 6.40% |  |
| Mixed | 36.80% | 59.90% | 38.40% | 60.90% |  |
| Other | 1.40% | 5.50% | 1.10% | 4.70% |  |
| Education |  |  |  |  |  |
| 0-7 years | 8.50% | 19.90% | 6.80% | 16.90% | p<0.01 |
| 8-11 years | 65.70% | 59.10% | 65.90% | 61.40% |  |
| 12+ Years | 25.70% | 19.20% | 27.10% | 20.30% |  |
| Other | 0.20% | 1.70% | 0.20% | 1.30% |  |
| **Hospital type** |  |  |  |  |  |
| Public | 36.00% | 47.90% | 37.00% | 48.30% | p<0.01 |
| Private | 24.40% | 17.60% | 23.60% | 17.70% |  |
| Mixed | 39.60% | 34.40% | 39.50% | 33.90% |  |
| **Municipality characteristics** |  |  |  |  |  |
| GDP per capita (thousand 2021 R$) | 62.70 (40.12) | 36.69 (28.10) | 60.32 (40.11) | 37.06 (36.77) | p<0.01 |
| Population covered by public primary healthcare | 38.18 (22.97) | 71.09 (25.81) | 38.16 (22.79) | 72.16 (25.52) | p<0.01 |
| Population with voluntary health insurance | 37.93 (13.09) | 17.81 (14.65) | 37.52 (13.00) | 17.45 (14.52) | p<0.01 |
| Public hospitals per beds per 100,000 inhabitants | 118.54 (73.63) | 156.63 (103.87) | 126.87 (76.41) | 166.56 (108.37) | p<0.01 |
| Physicians per 1,000 inhabitants | 2.61 (1.43) | 1.87 (1.40) | 2.89 (1.59) | 2.06 (1.52) | p<0.01 |
| Nurses per 1,000 inhabitants | 1.34 (0.56) | 1.18 (0.59) | 1.62 (0.69) | 1.48 (0.75) | p<0.01 |

Note: SP refers to São Paulo, OS refers to Other States. P-values are based on t-tests for continuous variables and chi-squared tests for categorical variables.

# Appendix B – Robustness checks: alternative covariate sets

| Figure B1 – Effects of the passage and withdrawal of Law 17,137 on c-section rates |
| --- |
| 1. Baseline: Model with no covariates |
| 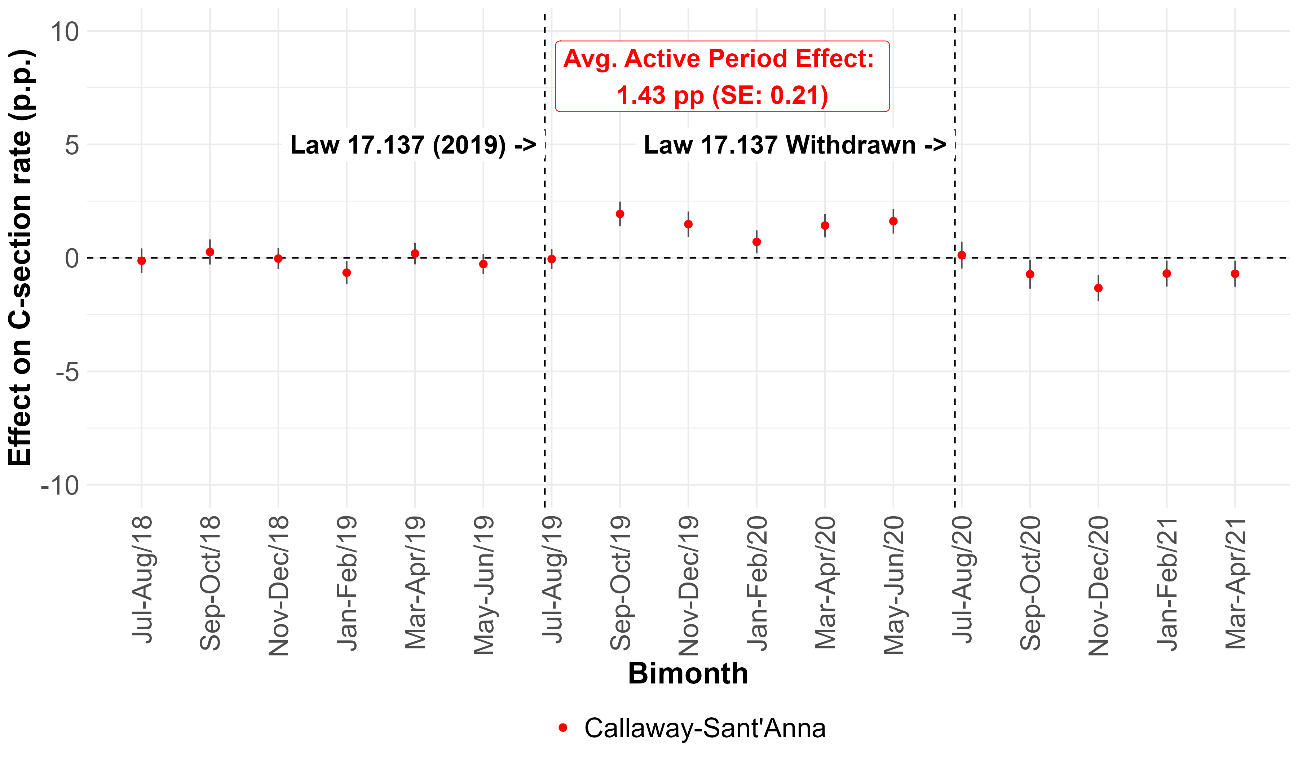 |
| 1. Model controling for maternal characteristics |
| 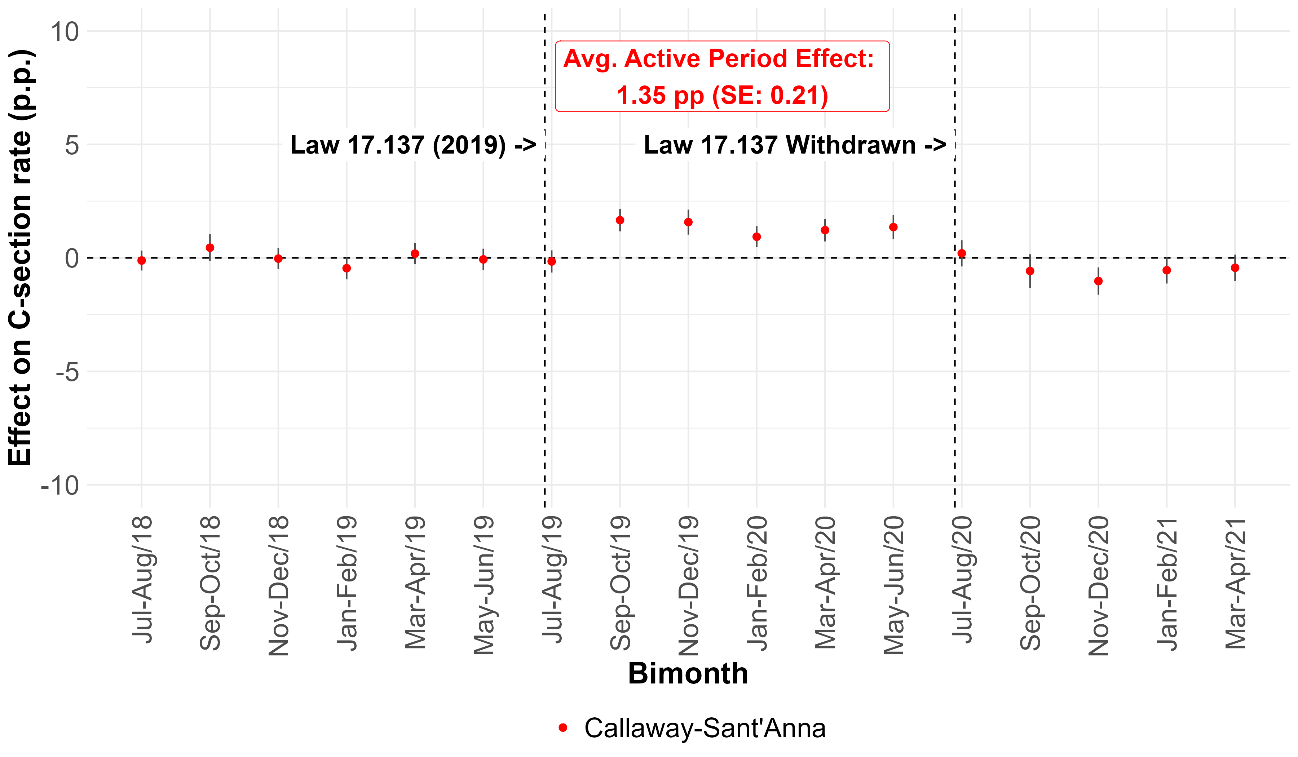 |
| 1. Model controling for municipal characteristics |
| 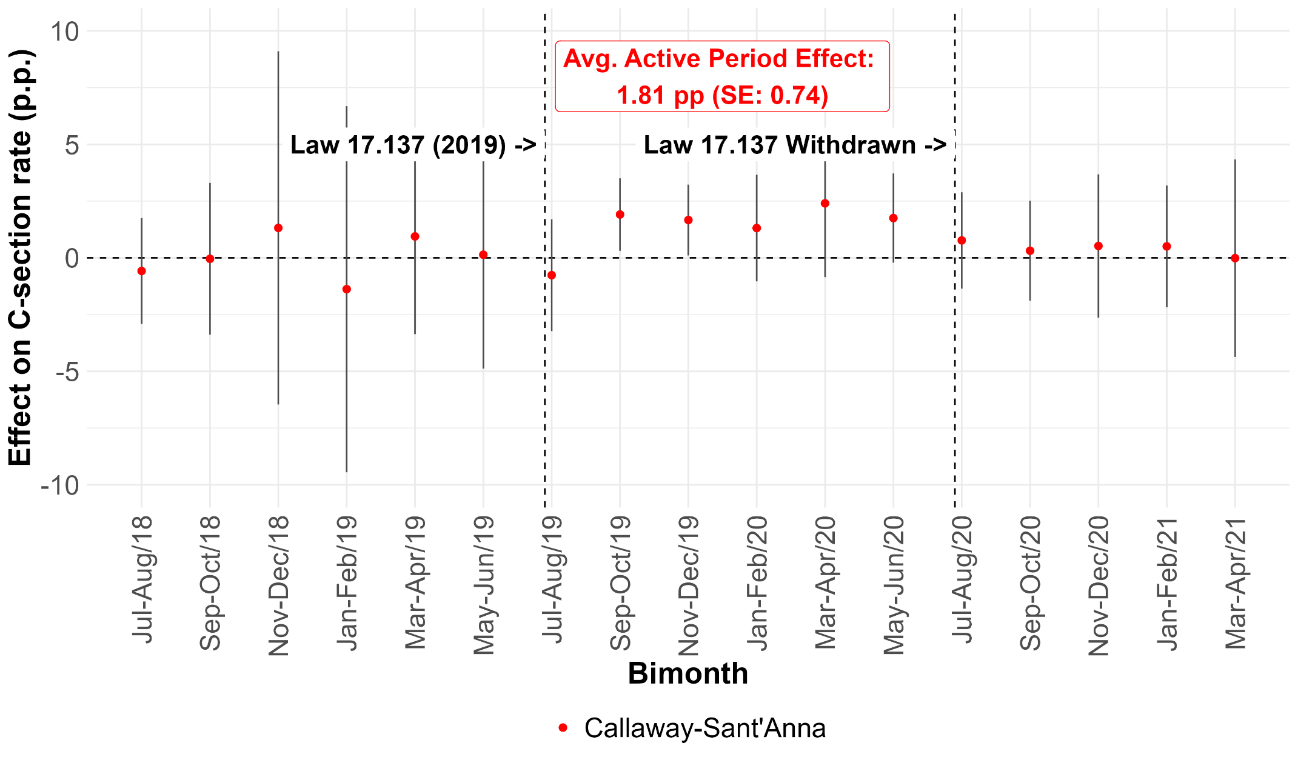 |
| 1. Model controling for both maternal and municipal characteristics |
| 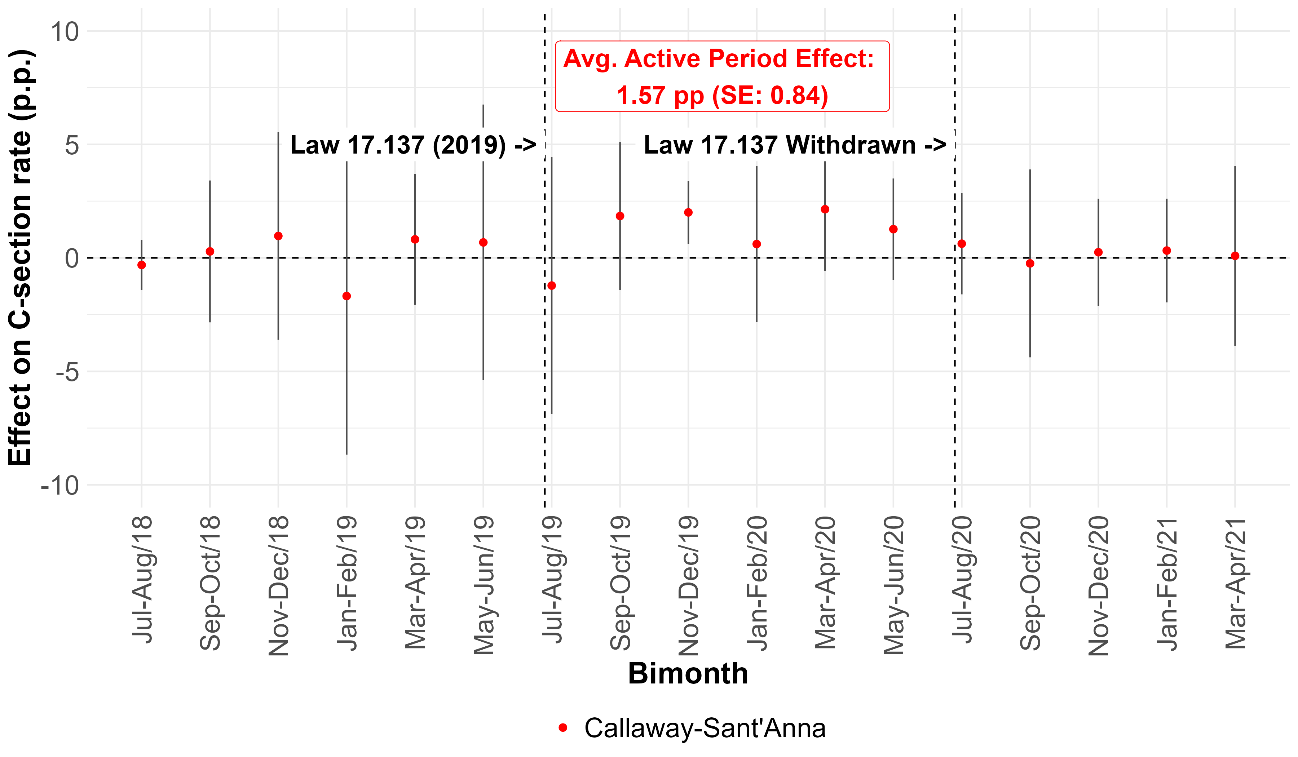 |

Estimates based on Callaway and Sant’Anna (2021)’s doubly-robust estimator. All models include time and municipality fixed effects. 95% Confidence Intervals based on standard errors clustered at the municipality level.

| Figure B2 – Placebo check – Placebo Law introduced one year earlier |
| --- |
| 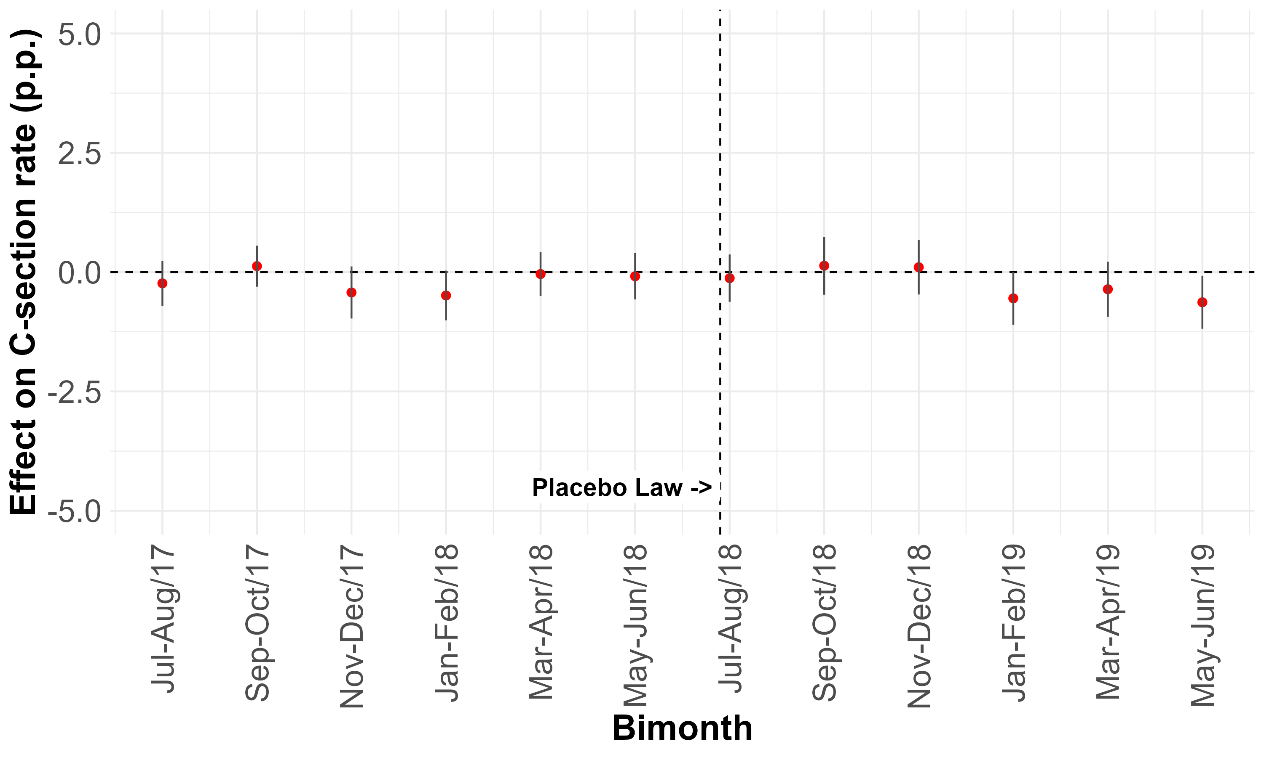 |

Estimates based on Callaway and Sant’Anna (2021)’s doubly-robust estimator. The model includes time and municipality fixed effects, but no other covariates. 95% Confidence Intervals based on standard errors clustered at the municipality level.

| Figure B3 – COVID-19 sensitivity check – Excluding the pandemic period |
| --- |
| 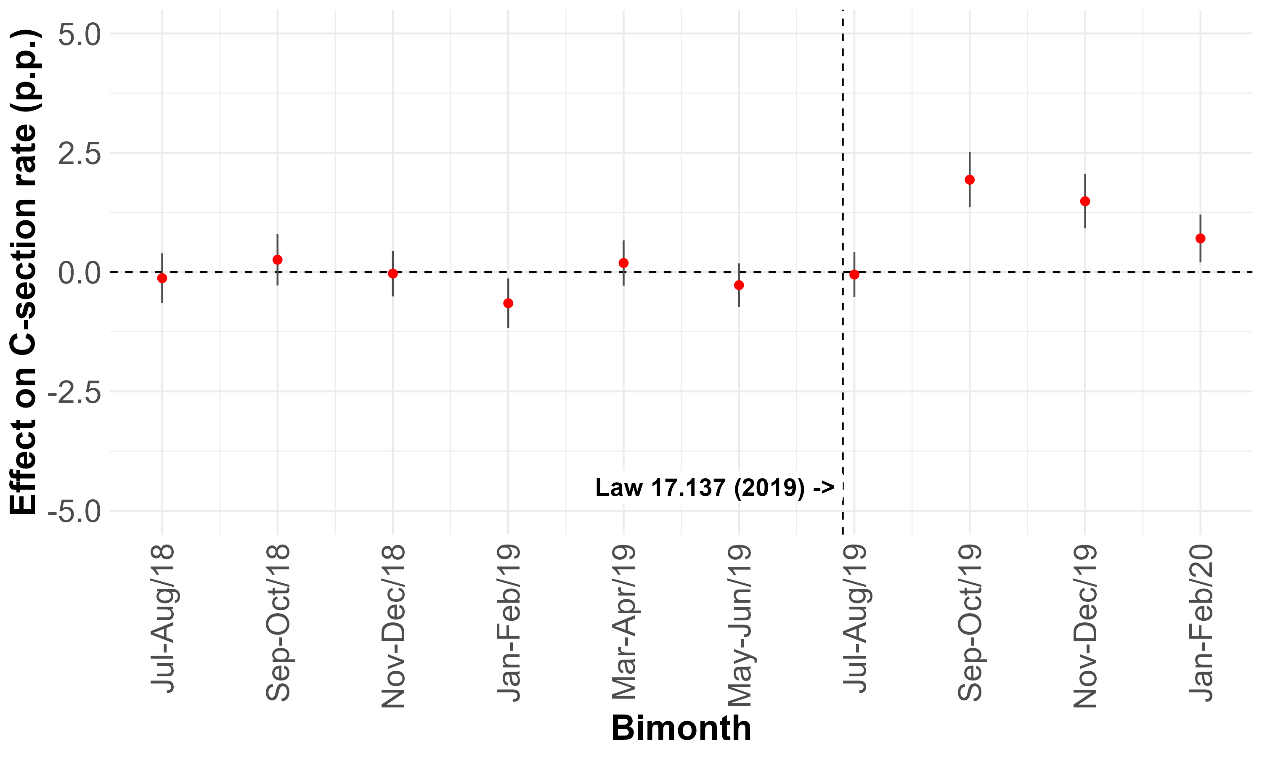 |

Estimates based on Callaway and Sant’Anna (2021)’s doubly-robust estimator. The model includes time and municipality fixed effects, but no other covariates. 95% Confidence Intervals based on standard errors clustered at the municipality level.

# Appendix C – Choice of hospital type for delivery

| Figure C1 – Effects of the passage and withdrawal of Law 17,137 on likelihood of birth in specific types of hospitals |
| --- |
| **PUBLIC HOSPITALS** |
| 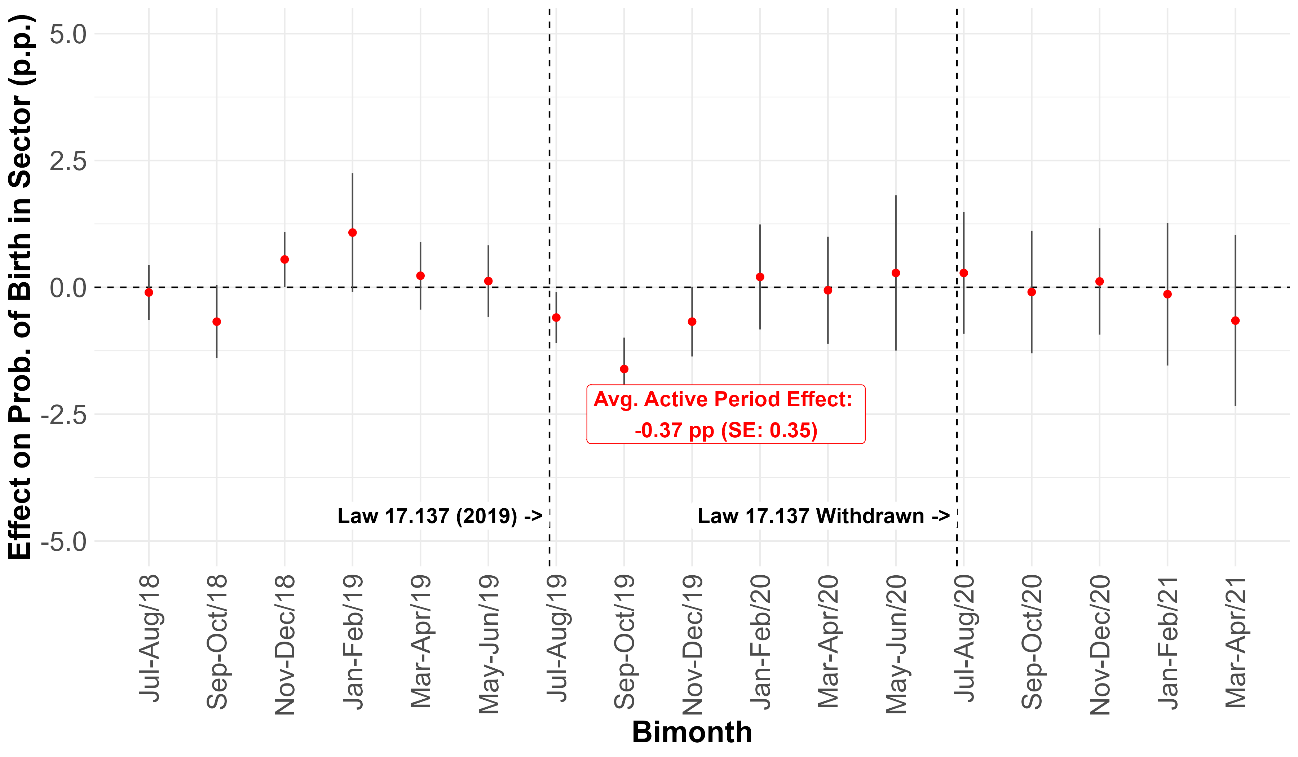 |
| **MIXED HOSPITALS** |
| 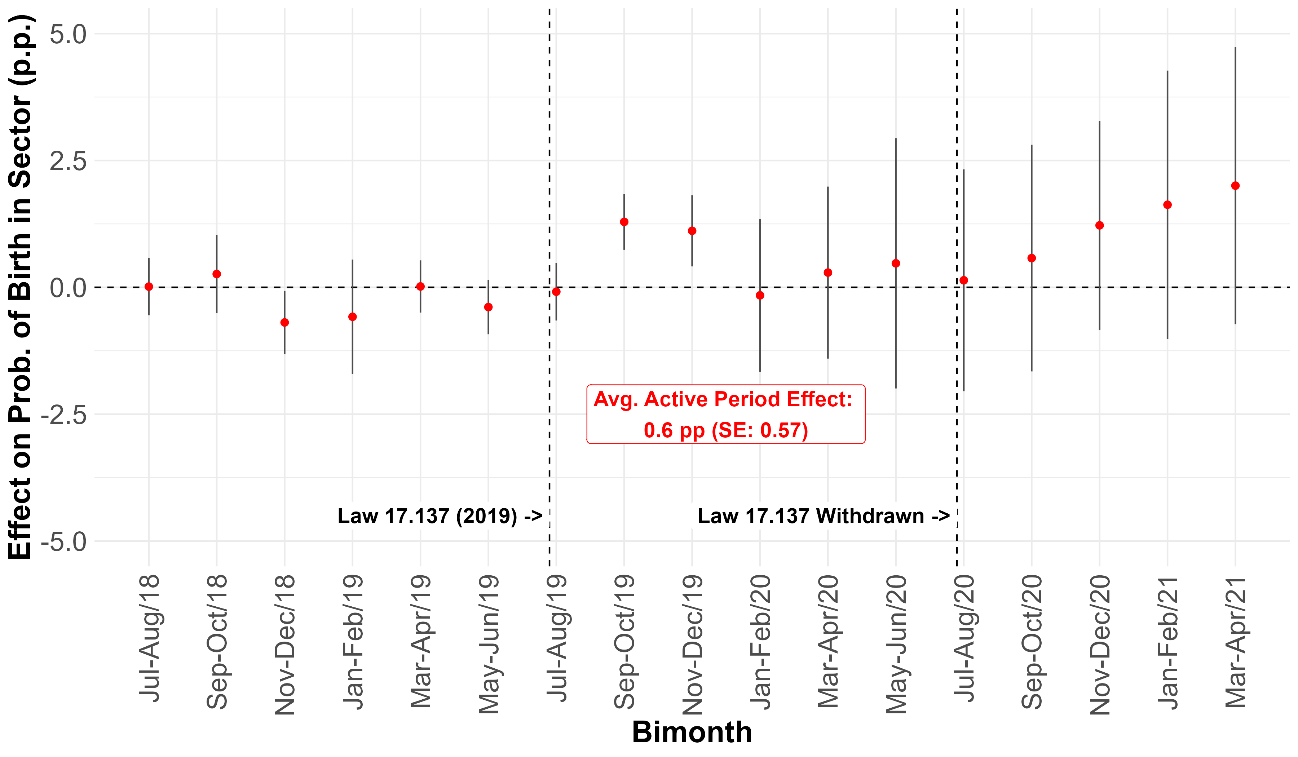 |
| **PRIVATE HOSPITALS** |
| 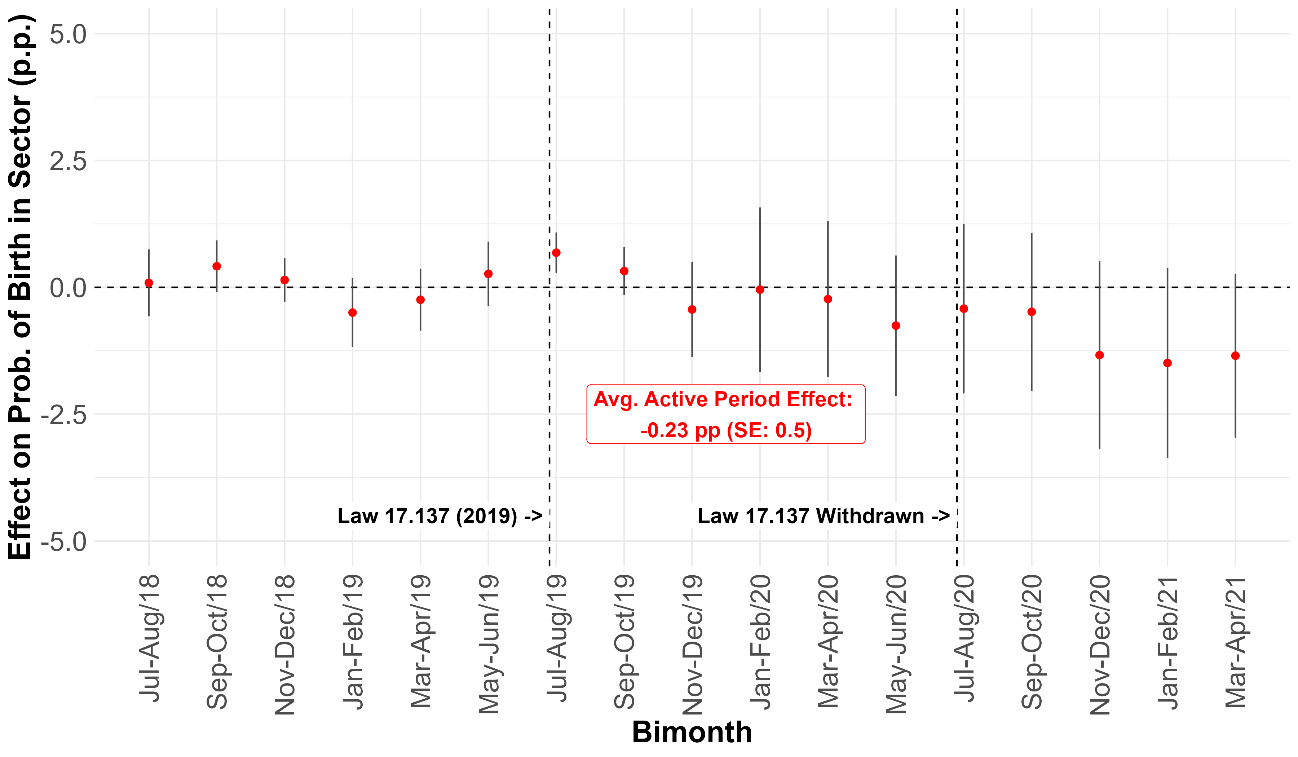 |

Estimates based on Callaway and Sant’Anna (2021)’s doubly-robust estimator. The model includes time and municipality fixed effects, but no other covariates. 95% Confidence Intervals based on standard errors clustered at the municipality level.

# Appendix D – Impacts on birth outcomes

| Figure D1 – Effects of the passage and withdrawal of Law 17,137 on neonatal health |
| --- |
| 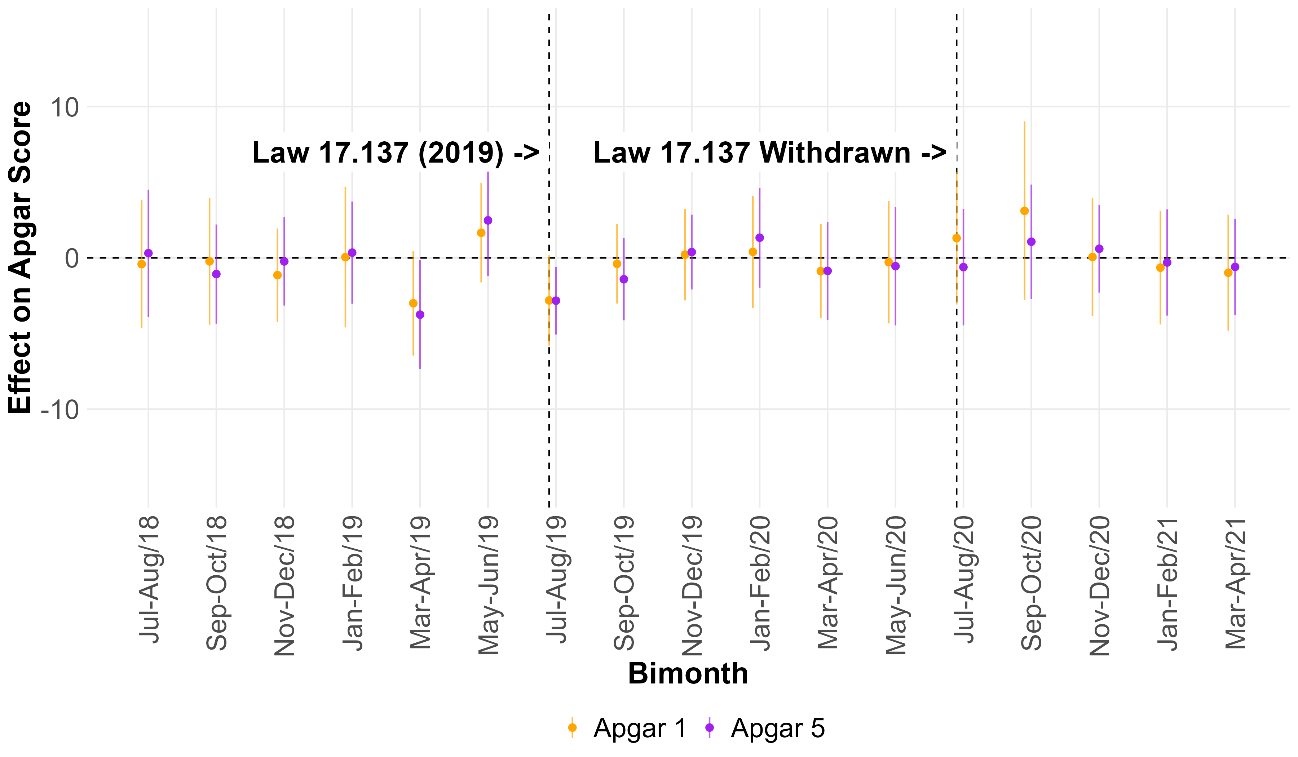 |

Estimates based on Callaway and Sant’Anna (2021)’s doubly-robust estimator. The model includes time and municipality fixed effects, but no other covariates. 95% Confidence Intervals based on standard errors clustered at the municipality level. Only births in public hospitals are included.

| Figure D2 – Effects of the passage and withdrawal of Law 17,137 on fetal development |
| --- |
| BIRTHWEIGHT |
| 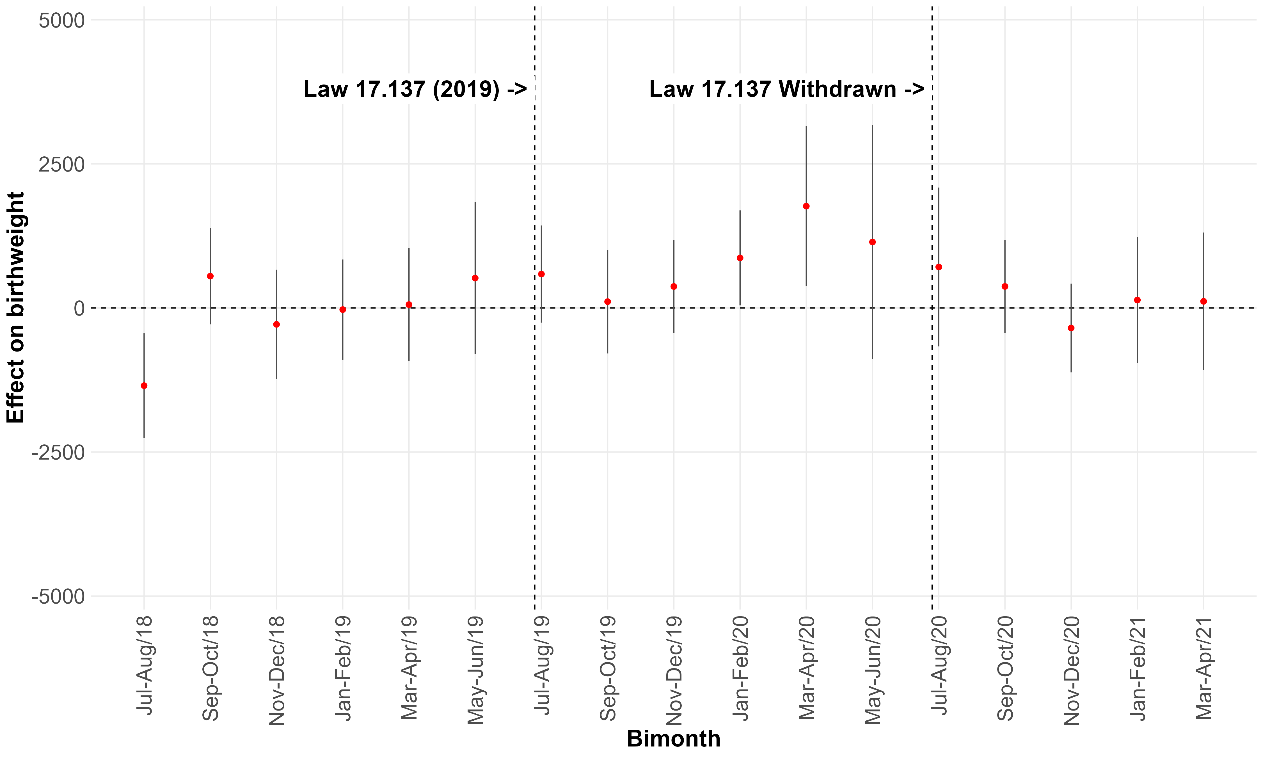 |
| GESTATIONAL WEEKS |
| 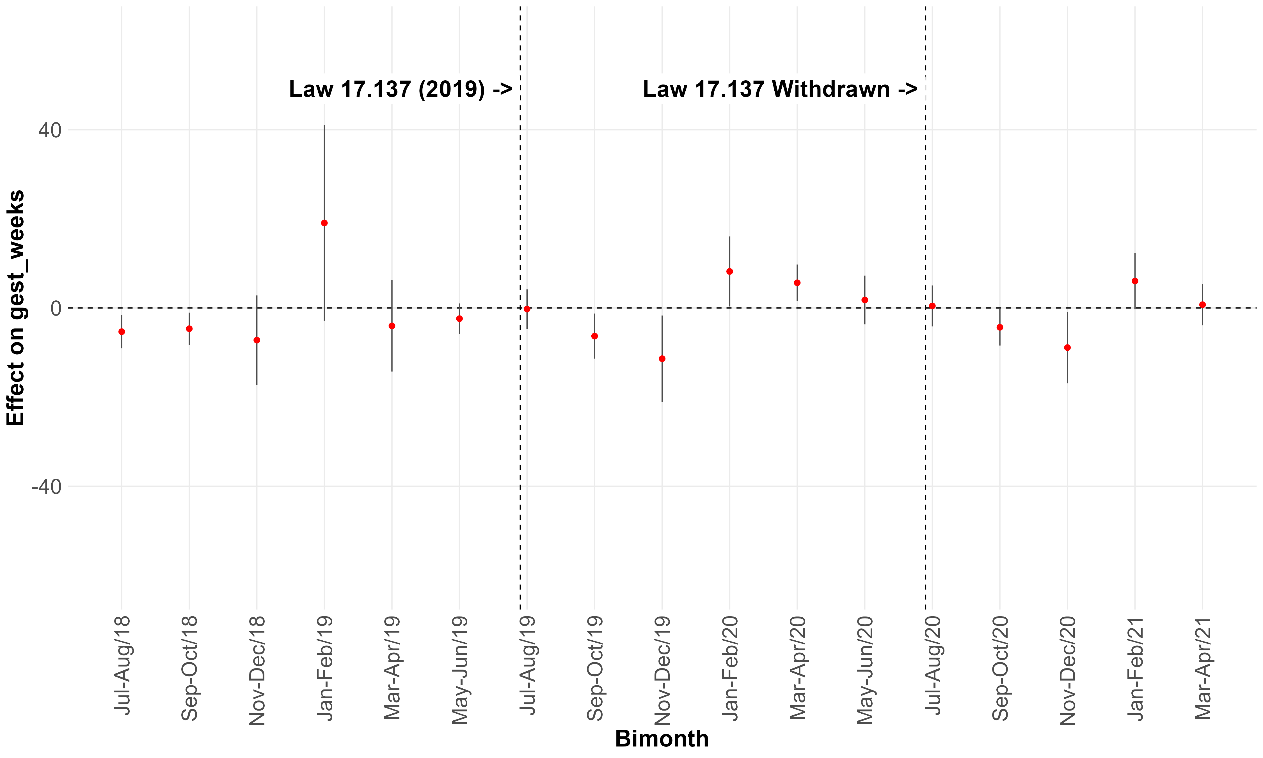 |

Estimates based on Callaway and Sant’Anna (2021)’s doubly-robust estimator. The model includes time and municipality fixed effects, but no other covariates. 95% Confidence Intervals based on standard errors clustered at the municipality level. Only births in public hospitals are included.

| Figure D3 – Effects of the passage and withdrawal of Law 17,137 on maternal and infant mortality and hospitalisation within 30 days of birth |
| --- |
| MATERNAL MORTALITY |
| 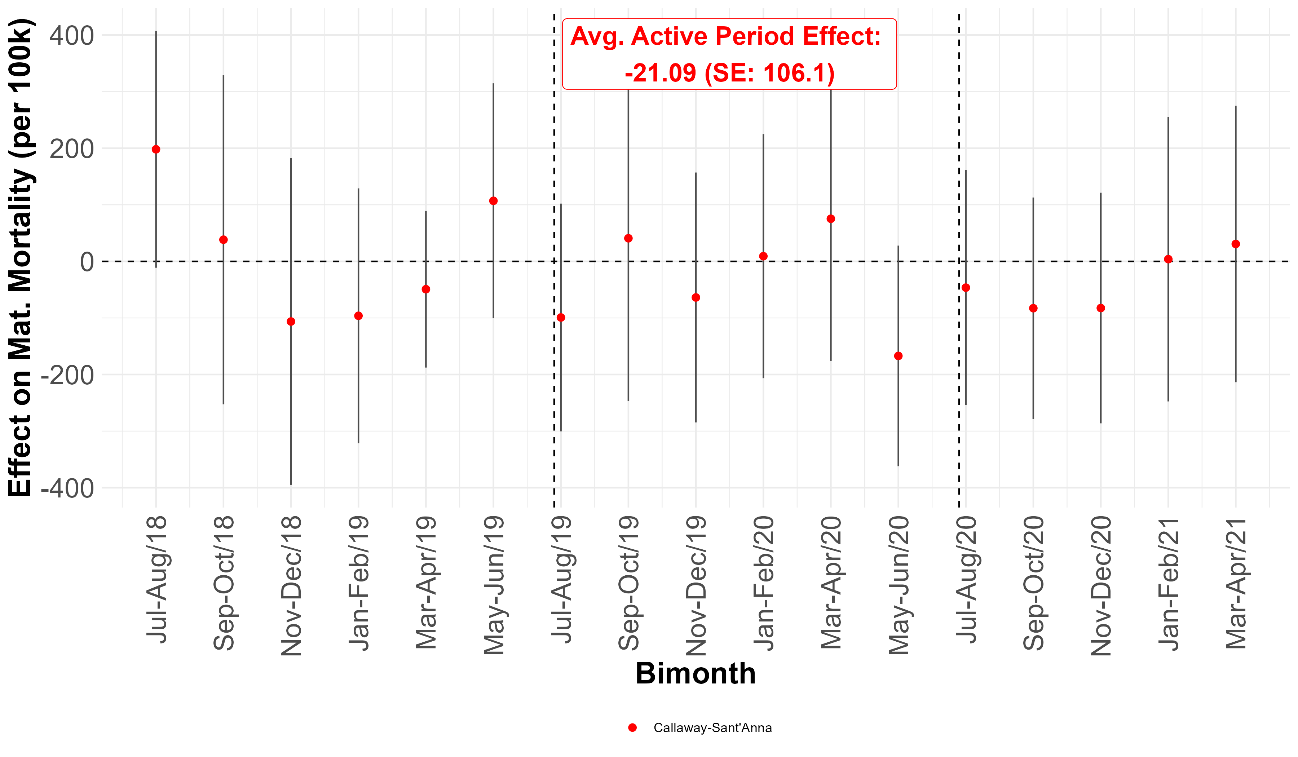 |
| 30-DAY INFANT MORTALITY RATES |
| 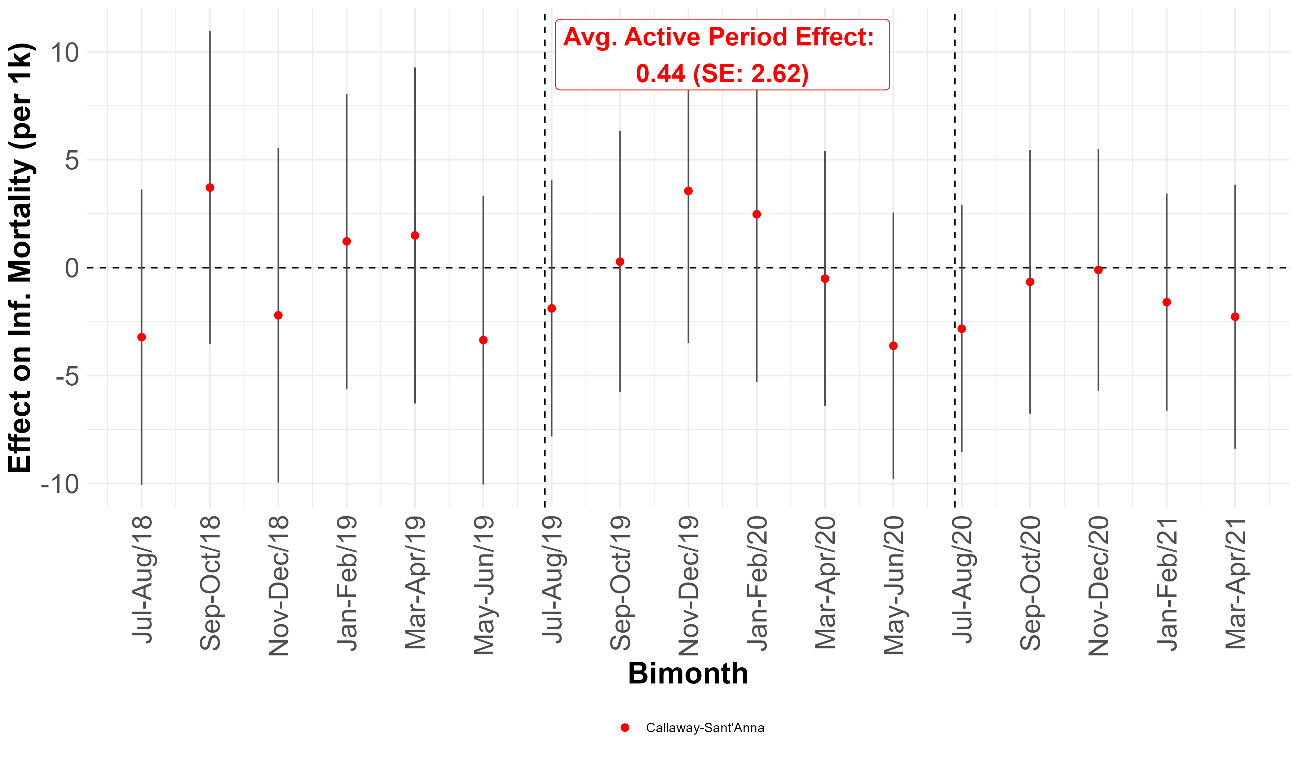 |
| INFANT HOSPITALIZATIONS WITHIN 30 DAYS OF BIRTH |
| 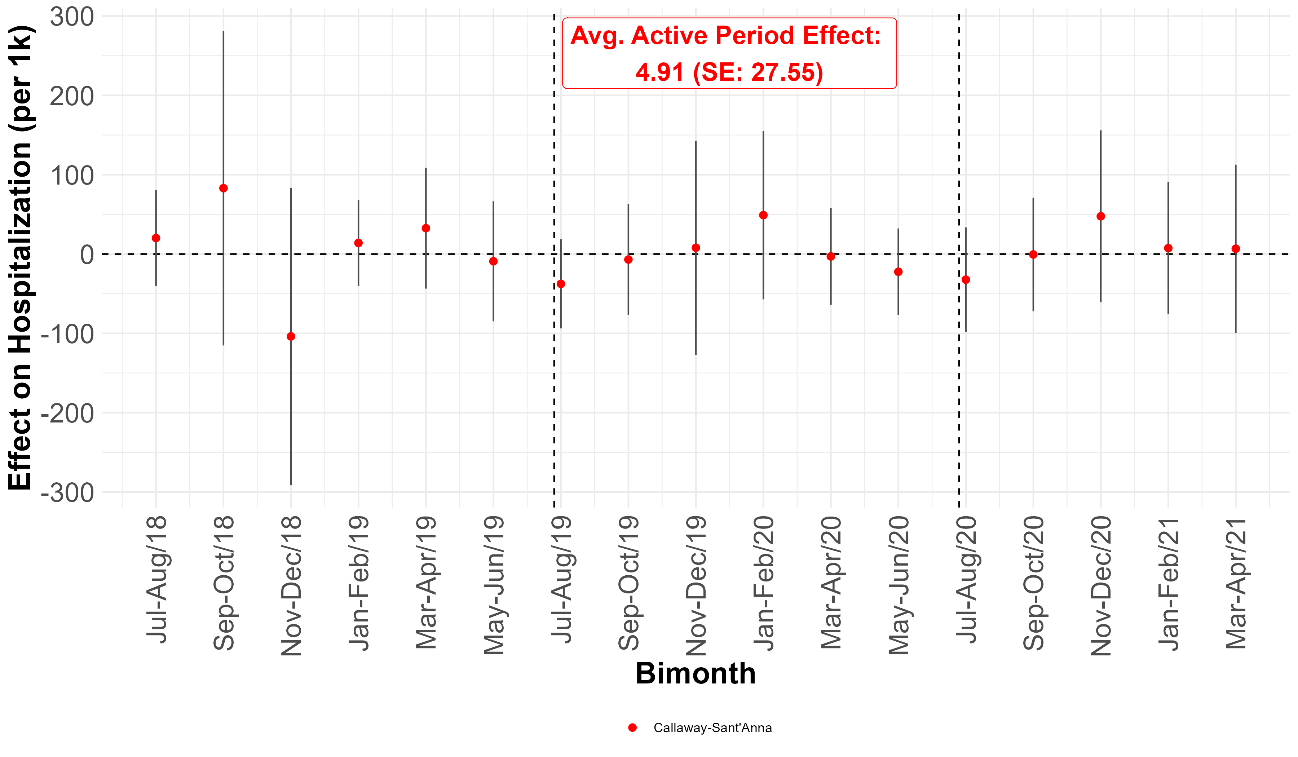 |

Estimates based on Callaway and Sant’Anna (2021)’s doubly-robust estimator. The model includes time and municipality fixed effects, but no other covariates. 95% Confidence Intervals based on standard errors clustered at the municipality level. Only births in public hospitals are included.

# Appendix E – Heterogeneity by municipal characteristics and demographic characteristics of mothers

| Figure E1 – Effects of the passage and withdrawal of Law 17,137 by baseline GDP per capita in the municipality |
| --- |
| 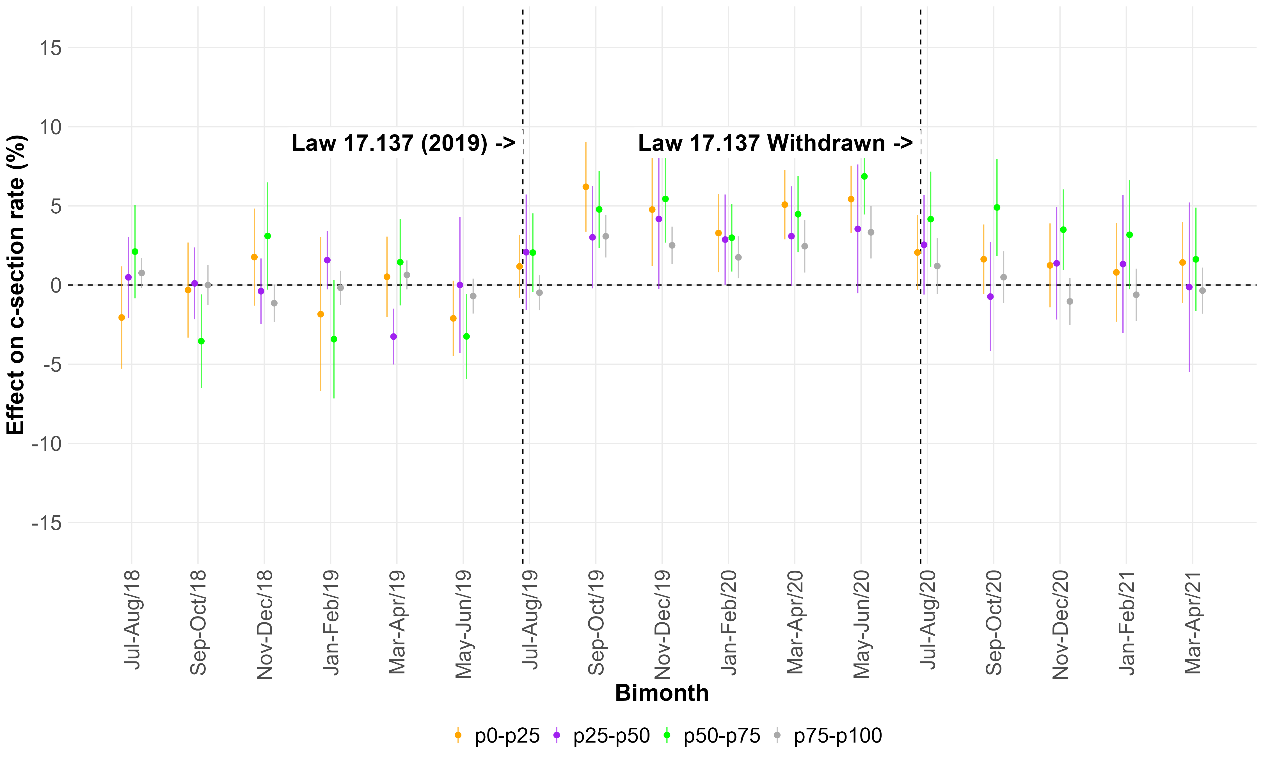 |

Estimates based on Callaway and Sant’Anna (2021)’s doubly-robust estimator. The model includes time and municipality fixed effects, but no other covariates. 95% Confidence Intervals based on standard errors clustered at the municipality level. Only births in public hospitals are included.

| Figure E2 – Effects of the passage and withdrawal of Law 17,137 by demographic groups |
| --- |
| EDUCATION LEVEL |
| 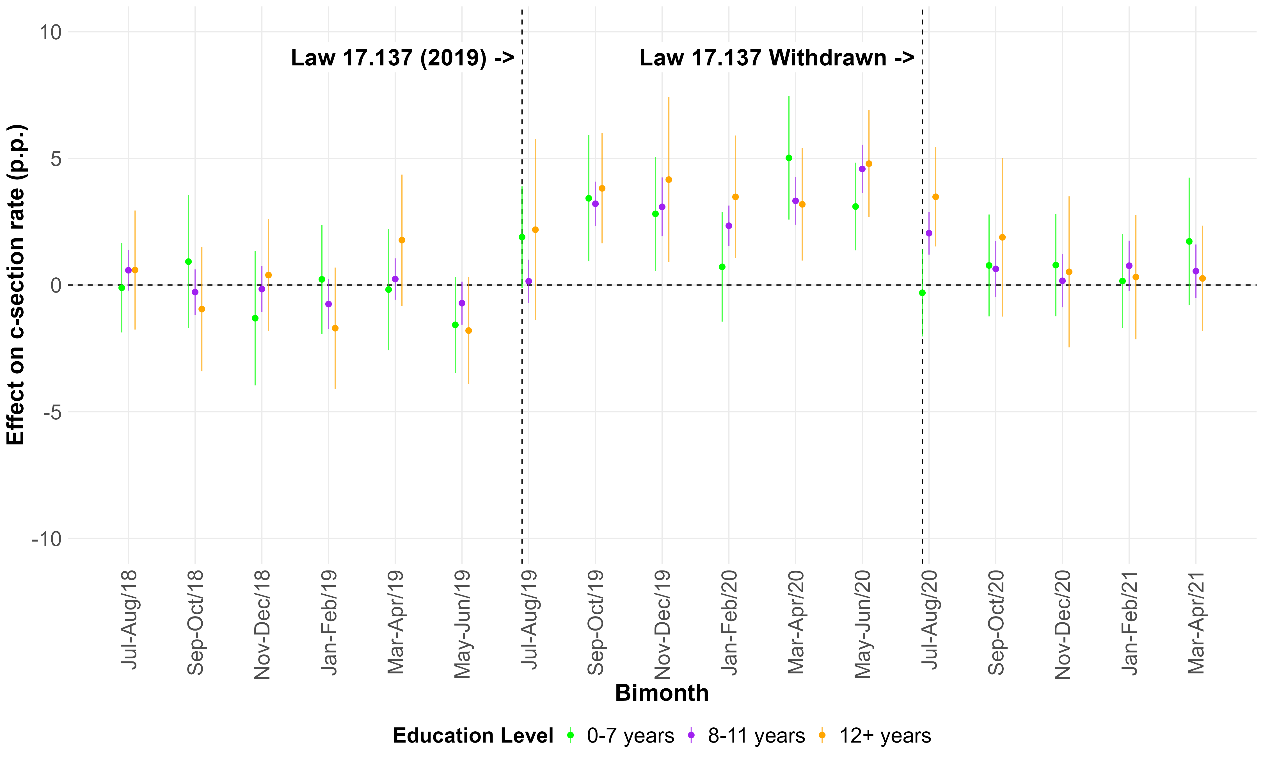 |
| RACE |
| 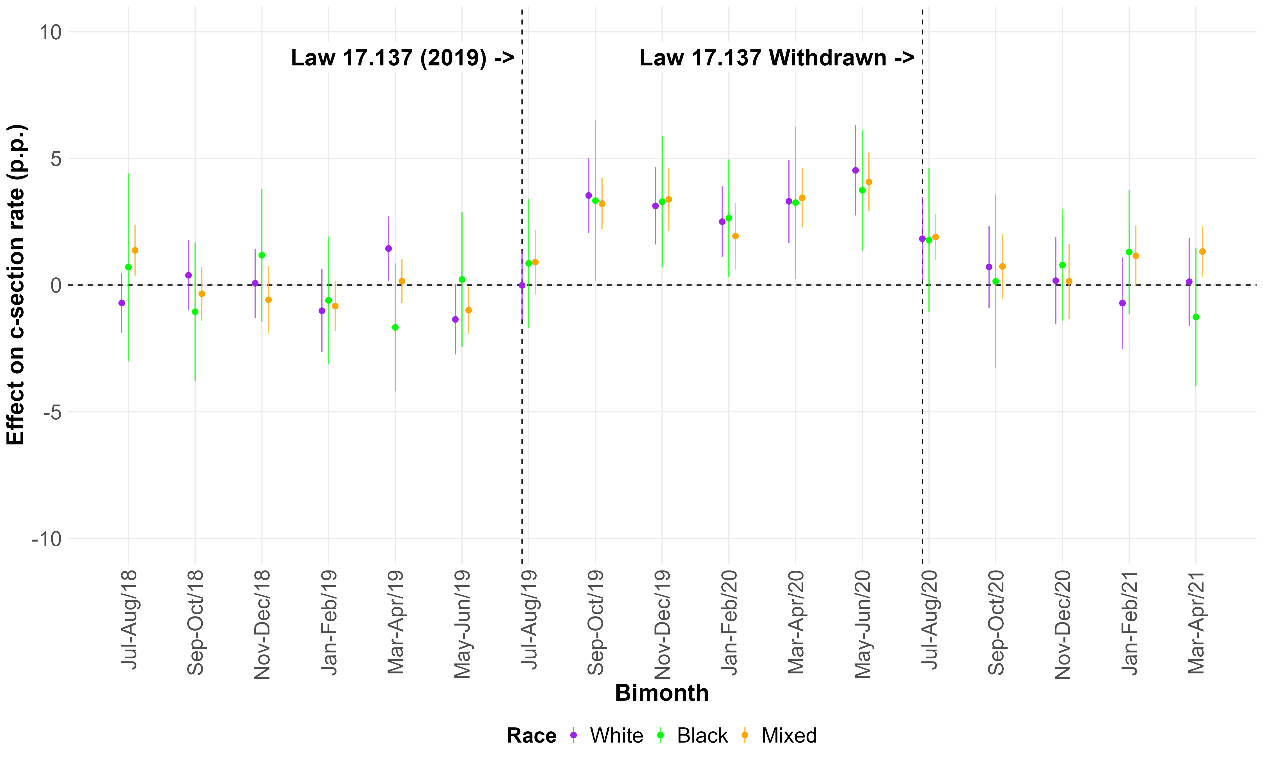 |
| MARITAL STATUS |
| 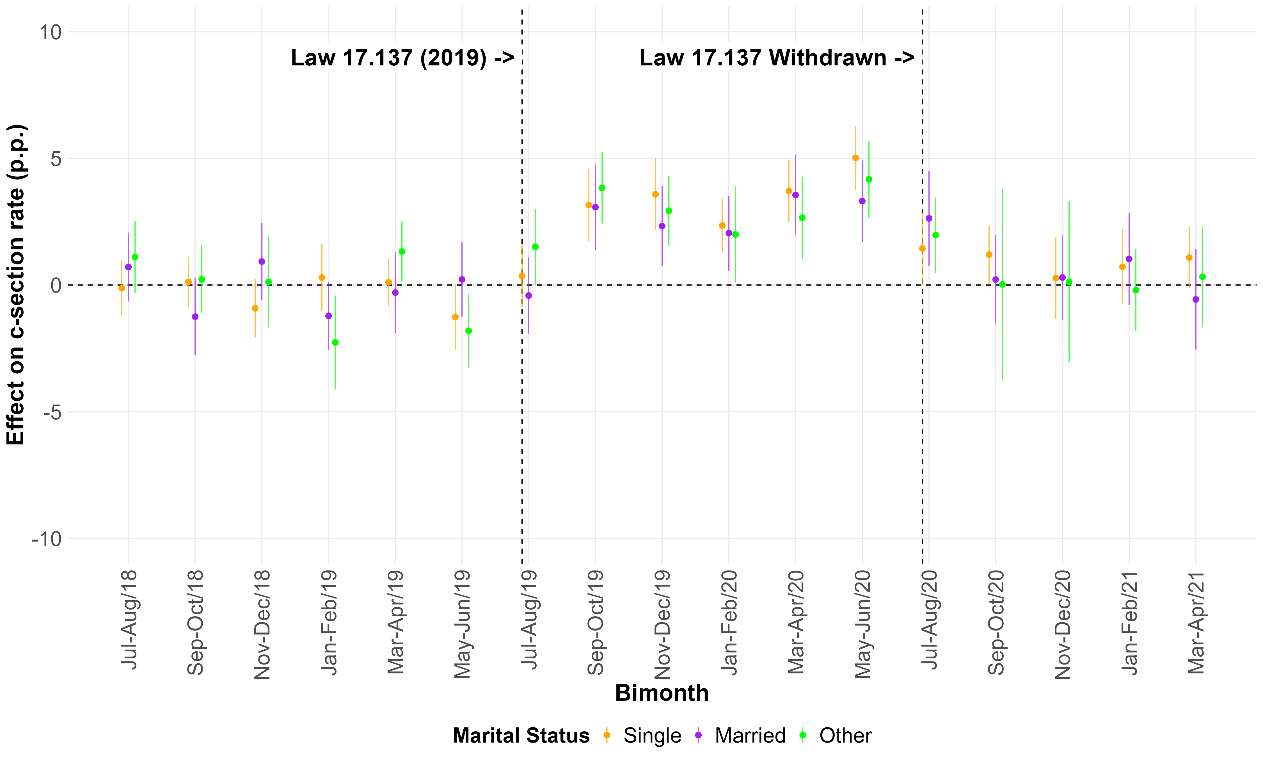 |

Estimates based on Callaway and Sant’Anna (2021)’s doubly-robust estimator. The model includes time and municipality fixed effects, but no other covariates. 95% Confidence Intervals based on standard errors clustered at the municipality level. Only births in public hospitals are included.
